# Systematic identification of cancer driving signaling pathways based on mutual exclusivity of genomic alterations

**DOI:** 10.1186/s13059-015-0612-6

**Published:** 2015-02-26

**Authors:** Özgün Babur, Mithat Gönen, Bülent Arman Aksoy, Nikolaus Schultz, Giovanni Ciriello, Chris Sander, Emek Demir

**Affiliations:** Computational Biology Center, Memorial Sloan Kettering Cancer Center, 1275 York Avenue, Box 460, New York, 10065 USA; Department of Epidemiology and Biostatistics, Memorial Sloan Kettering Cancer Center, 1275 York Avenue, New York, 10065 USA; Tri-Institutional Training Program in Computational Biology and Medicine, 1275 York Avenue, New York, 10065 USA

## Abstract

We present a novel method for the identification of sets of mutually exclusive gene alterations in a given set of genomic profiles. We scan the groups of genes with a common downstream effect on the signaling network, using a mutual exclusivity criterion that ensures that each gene in the group significantly contributes to the mutual exclusivity pattern. We test the method on all available TCGA cancer genomics datasets, and detect multiple previously unreported alterations that show significant mutual exclusivity and are likely to be driver events.

**Electronic supplementary material:**

The online version of this article (doi:10.1186/s13059-015-0612-6) contains supplementary material, which is available to authorized users.

## Background

Only a small fraction of genomic alterations present in a tumor are selected directly because of their ability to increase cellular proliferation and to unlock barriers against growth and metastasis. The majority of the observed alterations, the so-called passengers, are indirectly selected due to incidental co-occurrence with a driver alteration or other selected event [1]. Differentiating drivers from passengers in cancer can help us to identify tumorigenic mechanisms and drug targets, and to design patient-specific therapeutic interventions.

**Figure 1:**
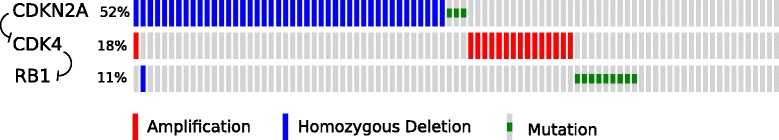
**Distribution of CDKN2A, CDK4 and RB1 mutations and copy number changes.** These are from the The Cancer Genome Atlas (TCGA) glioblastoma dataset, as provided by cBioPortal. At least one of the genes is altered in 78% of the cases, with an overlap in only two samples. Even though RB1 is mutated only in 11% of the cases, its activity is potentially affected by alterations of the other two genes, which encode for upstream proteins in the signaling network.

Pivotal driver events, such as TP53 loss-of-function mutations, can be identified simply by their significantly high alteration rate in a set of tumors. More often, however, not one but several alternative driver alterations in different genes can lead to similar downstream events. In those cases, the selection bonus is divided among the alteration frequencies of these genes. For current cancer genomics studies where the number of samples is two orders of magnitude smaller than the number of profiled genes per sample, the statistical power of naive frequency-based methods is not sufficient to differentiate these substitutive drivers from passengers (Figure 1).

A key observation is that when a member of a substitutive set is altered, the selection pressure on the other members is diminished or even nullified. As a result, we expect significantly less overlap in alterations of the alternative driver genes, creating a mutual exclusion pattern between their alterations. Supporting this expectation, it was previously shown that some functionally related genes are altered mutually exclusively in thyroid tumors [2,3] and in leukemia [4].

This principle was first applied systematically by Yeang et al. to detect substitutive driver groups in cancer [5]. Their method calculates all pairwise mutual exclusion relations with a hypergeometric test. Miller et al. improved this approach by developing a statistical significance measure for the modules identified via pairwise exclusivity [6]. Ciriello et al. use a protein interaction graph for searching sets of mutually exclusive gene alterations [7]. They test each clique in the interaction network by random permutations to see if the observed overlap is significantly small. By using prior interaction knowledge, this approach can dramatically limit the search space. Vandin et al. suggest a weight function to score mutually exclusive alterations, which rewards coverage (number of samples altered in at least one of the genes in the group) while penalizing overlap [8]. They, then, search for subsets of genes that maximize the weight function. Zhao et al. and Leiserson et al. use the same weight function and expand on the search technique [9,10]. Szczurek et al. propose a generative model for mutual exclusivity and test if the observed distribution of alterations fits this model better than a random model [11]. Their generative model assumes that genes in a module have an equal chance of being altered, hence their result modules typically contain genes with similar alteration ratios.

We are expanding on these approaches by combining detailed prior pathway information with a novel statistical metric to improve both accuracy and biological interpretation and to validate the results. Specifically, we are using a large aggregated pathway model of human signaling processes to search groups of mutually exclusively altered genes that have a common downstream event. To enable this search, we also define a new statistical test that satisfies the following criteria:

1. Soft: There are two kinds of mutual exclusivity defined in the statistical literature: hard and soft. Hard mutual exclusivity [12] tests for two events that are assumed to be strictly mutually exclusive and the null hypothesis is that overlaps between them can be explained by random errors. The biological mechanism we are testing for, however, should lead to soft mutual exclusivity where two otherwise independent events overlap less than expected by chance because of a statistical interaction – in this case *partially* overlapping selective advantages.
2. Analytical: Scoring the mutual exclusion of a set of alterations with random permutation testing is computationally expensive. Ciriello et al. use such a metric for detecting significant cliques because the number of tested cliques is limited. In a wider search that includes non-clique subgraphs, such as upstream signaling pathways, the number of hypotheses that need to be tested increases by several orders of magnitude, making the permutation testing infeasible. We, therefore, need an analytical test that can scale up to large datasets and a very large number of searches.
3. *N*-ary: For two genes, soft mutual exclusivity can be measured analytically with the hypergeometric test, also known as Fisher’s exact test. There is no consensus, however, for testing the mutual exclusivity of more than two genes analytically. Yeang et al. test whether all pairwise interactions are significant in the group. This, however, is an overly strict test as a gene set can exhibit a strong mutual exclusion pattern as a group even if none of the pairs are significantly mutually exclusive. The weight function used in Vandin et al., Zhao et al. and Leiserson et al. can test arbitrarily large sets. This metric, however, has a strong bias toward highly altered genes, and in some cases can select randomly occurring high coverage and high overlap sets, resulting in both false positives and negatives. We provide examples for these cases in the fourth section of Additional file 1. Our proposed metric is an extension of the hypergeometric test to quantify the mutual exclusivity between more than two measurements while retaining the analytical and soft properties.

Our scoring metric can be applied to a wide spectrum of searches with or without the prior information. In both types of scenarios, it compares favorably to the existing methods (see Results). In this manuscript, we focus on searching for mutually exclusively altered groups where members have a common downstream signaling target as defined in the public pathway databases. The rationale behind this is to limit the search space to a subregion that has a higher density of true positives. At the cost of some recall, this reduction mitigates the statistical power loss due to multiple hypothesis testing. Another advantage of this reduction is that it nominates a preliminary mechanistic explanation for the observed mutual exclusivity – specifically a common effect on a downstream gene. It is, however, important to note that this is just one potential mechanism out of many – it should be treated as a starting point for further biological inquiry. The statistical significance of the observed mutual exclusivity is independent of the hypothetical mechanism that nominated it for testing.

We tested our method on 17 different cancer datasets from The Cancer Genome Atlas (TCGA) in cBioPortal [13], and identified multiple significantly altered gene groups that are functionally related. We also present a comparison of the performance of existing methods on simulated datasets.

## Results

### Measure for mutual exclusion

**Figure 2:**
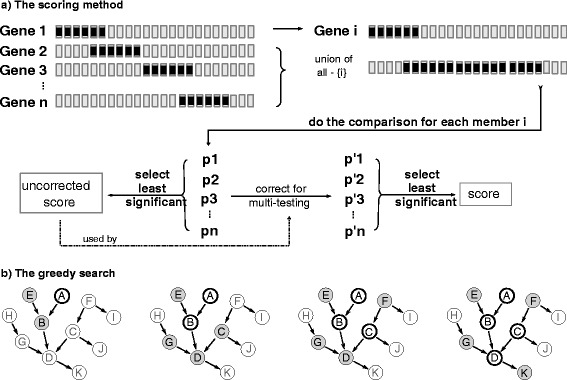
**Searching and scoring method.** **(a)** Mutual exclusivity of a group of gene alterations is evaluated by comparing each gene with the union of the other genes. The initial score is the least significant *P* value. To correct for multiple hypothesis testing, we estimate the significance of the initial *P* values during a search with permuted alterations. The least significant of these second *P* values is the multiple hypothesis testing corrected group score. **(b)** At each step of the greedy search, we expand the group with the next best candidate gene from the surrounding genes that have a common downstream target with the group members, or they are a common downstream target themselves. In this illustration, four sample steps of the search are shown for a sample network. Thick-bordered genes are current group members and genes with a gray background are candidates for the next expansion. The best-scoring candidate gene is added to the group if it increases the score, and the candidates are re-assessed for the next phase. The search will stop if the group cannot expand anymore or a threshold group size is reached.

To measure mutual exclusion of a group of genes, we test each gene against the union of all other alterations in the group, and use the least significant *P* value as the initial score of the group. To correct for multiple hypothesis testing, we estimate the member genes’ probability to have the observed *P* value in a result group by chance, and derive the corrected *P* values. We use the least significant of this second set of *P* values as the final score of the group (Figure 2a, also see Methods).

### Searching the mutually exclusive group

We built a large, directed gene network collecting interactions from the Pathway Commons [14], SPIKE [15] and SignaLink [16] databases. This network is available in the Additional file 2 archive and its generation was described previously [17]. We use this network to search for groups of mutually exclusive genes that have a common downstream target in the network. We start the search by initializing a group with an altered gene as the seed of the group, and greedily expand with the next best candidate gene. We define candidate genes such that after addition of a candidate, the members will still have a common downstream gene that can be reached without traversing any non-member genes (Figure 2b). Note that a common downstream gene can also be a member. We greedily expand the group with the candidate that best improves the group score. The search terminates when there are no remaining candidates or when the group size reaches a preset threshold. The algorithm returns a group and its score for each seed gene. To control the false discovery rate (FDR) in the resulting groups, we estimate the null distribution of the final scores by running the same analysis on a set of permuted datasets, where gene alteration ratios and network connectivity are preserved, but sample distributions of alterations are shuffled.

We used this algorithm to identify mutual exclusion in the mutation and copy number profiles from 17 TCGA studies [18-25], deposited in cBio Portal [13]. We cropped the gene network to the proximity of significantly mutated genes (provided by MutSig [26]) and copy number significantly altered genes (provided by Gistic [27]). Here, proximity means the neighbor genes and the genes that have a common downstream target. Thresholds of 2 and −2 were used for copy number amplification and deletion, respectively, for discretized Gistic values. We used only the copy number changes that are confirmed by an expression change (see Materials and methods). For each study, we filtered out genes that have a low alteration rate to reduce the noise in the data. We searched groups up to size 5. We ran 10,000 permutations for estimating the null distribution of the member *P* values of genes in groups, and we used 100 iterations for estimating the null distribution of the final group scores in the result. For each study, we selected the FDR cutoff that maximizes the expected value of *true positives* −*false positives* in the results.

The distribution of alterations in endometrial cancer samples is exceptional in the sense that samples are strongly dominated by either copy number alterations or mutations (Additional file 1: Figure S1). Because of this, many of the copy number changes are mutually exclusive with many of the mutations. Since mutual exclusion of the copy number alterations and mutations result from a higher order event, the mutual exclusivity for these two subtypes have little additional biological implication. To remove the effect of these highly distinct subtypes, we divided the endometrial cancer samples into two, and treated them as different studies.

### Results for TCGA datasets

**Figure 3:**
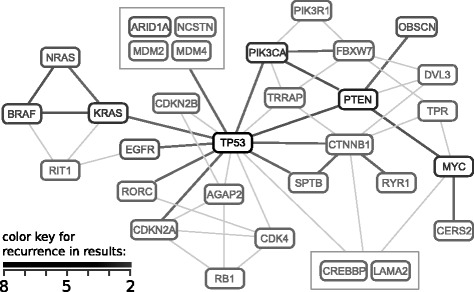
**Network constructed using recurrent genes in the results.** The genes in the graph appear in the results of at least two different studies. Thick edges represent recurrent co-presence of gene pairs in the same mutually exclusive set. Thin edges represent non-recurrent co-presence of gene pairs, and are only used to connect the genes that lack a recurrent edge. Note that there are many other non-recurrent edges between recurrent genes, which are omitted from the graph to reduce complexity. See Additional file 1: Figure S44 for a complete graph with all non-recurrent genes and co-presences.

This section contains an overview of the results, while we provide the detailed analysis results of individual studies in Additional file 1. There are a total of 199 genes in Mutex results, and we observe that 31 genes appear in the results of at least two studies. We also observe that many gene pairs are recurrently co-present in the result groups (Figure 3). Not surprisingly, the most recurrent gene in the results is *TP53*, followed by *PTEN*, *KRAS*, *MYC*, *PIK3CA*, *BRAF*, *EGFR* and *NRAS* – all well-known tumor suppressors or oncogenes. The two next most recurrently found genes are *OBSCN* and *ARID1A*. *OBSCN* functions in myofibrillogenesis, and is known to activate Rho GTPases [28,29]. Motif-based studies also predict that it binds to PIK3R1 – the regulatory component of the PI3K complex [30]. *ARID1A* was previously shown to be a tumor suppressor in gastrological cancers.

To demonstrate the novelty of our results, we searched for co-citation of the recurrent genes with the word ‘cancer’ in the literature using CoCiter [31] (Additional file 1: Table S2). We observe that the last ten genes in the list (*TRRAP*, *AGAP2*, *CERS2*, *RORC*, *NCSTN*, *LAMA2*, *RIT1*, *OBSCN*, *RYR1* and *SPTB*) have less than 25 co-citations, while well-known genes have hundreds or thousands of co-citations. Since these ten genes are not well-known cancer drivers, we looked for other evidence that supports their cancer relevance; specifically if they are recurrently mutated or copy number recurrently altered enough to be detected by MutSig or Gistic, respectively, and checked whether they contain mutation hotspots (Additional file 1: Table S2). MutSig can detect *RIT1* in lung adenocarcinoma and *OBSCN* in adrenocortical carcinoma. Gistic can detect nine of these genes in different cancers, but note that Gistic results are considered to be very weak evidence because copy number alterations generally affect large portions of the chromosomes, and most of the recurrent copy number changes are considered to be passengers. For the total of 17 studies, Gistic reports 13,123 genes using 5% FDR cutoff, while Mutsig reports only 388.

Mutation hotspots are considered to be evidence of driver mutations, because mutations in different parts of a driver gene are likely to bring different amounts of selective advantage to a cancer cell, while passenger mutations are expected to be randomly distributed. Of these ten less-known genes, we find that five of them (*TRRAP*, *OBSCN*, *RIT1*, *AGAP2* and *RORC*) contain mutation hotspots (Additional file 1: Figure S45). Note that among the remaining five genes, two of them (*CERS2* and *NCSTN*) are mostly copy number altered in the results. A literature survey of these ten genes helps us understand their cancer context, as we summarized in Table 1.

**Table 1:** **Ten recurrent genes that are least associated with cancer in the literature**

**Gene**	**Possible relevance for cancer**
*TRRAP*	This is a member of the PIK-related kinases family, and previously it was suggested it mediates transcriptional control of TP53 on *MDM2* [32]. We find *TRRAP* in result groups both with *PIK3CA* and with *TP53* for two subtypes of endometrial cancer.
*AGAP2*	Known to be over-expressed in cancer cells, and it was suggested it carries anti-apoptotic signals by activating nuclear phosphoinositol 3 kinases [33].
*CERS2*	A ceramide synthase. Ceramide was previously classified as a tumor-suppressor lipid [34].
*RORC*	This is a nuclear receptor and it was previously associated with secondary lymphedema formation after breast cancer surgery [35].
*NCSTN*	This is a component of the gamma-secretase complex, which cleaves many target proteins including Notch and Ecadherin. In a study, it was found to be over-expressed in about half of breast cancer cells, and its knock-down was shown to reduce cell invasion [36].
*LAMA2*	The alpha subunit of laminin. It functions in cell attachment and mobility. It is also known to function in a complex that activates Rho GTPases [37].
*RIT1*	This is a Ras-related GTPase, and is involved in the Ras-MAPK signaling cascade. Its mutations were recently classified as driver for lung adenocarcinoma cells [38].
*OBSCN*	This gene functions in myofibrillogenesis, and is known to activate Rho GTPases [28,29].
*RYR1*	Ryanodine receptors are calcium release channels found in skeletal muscle and neuronal cells. It was previously reported that RyR expression occurs frequently in breast cancer and correlates with tumor grade [39].
*SPTB*	This is a member of the spectrin family, which are membrane cytoskeletal proteins that function in cell membrane organization and stability. It was previously shown that another spectrin, SPTBN1, functions in TGF-beta signaling, and its loss can contribute to hepatocellular cancer [40].

**Figure 4:**
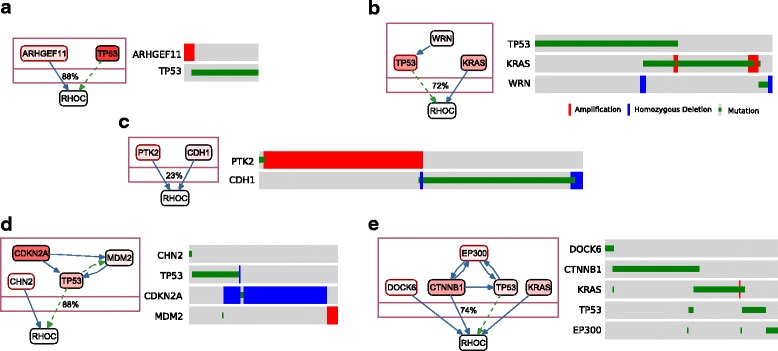
**Sample result groups that have RHOC as a common downstream target.** Oncoprints are shown at the right of each group and unaltered samples are omitted. Gene color intensities are proportional to gene alteration ratios. In the signaling network, dashed green edges represent transcriptional relations and solid blue edges represent post-translational relations. Genes in the result groups are shown inside a compound node, whose label shows the alteration coverage of samples in the group. This is also equal to the visible portion of samples on the oncoprints. Figure prepared using ChiBE [43,44]. **(a)** Uterine corpus endometrial carcinoma (CNA dominated 56 samples, 49 altered). **(b)** Lung adenocarcinoma (218 samples, 160 altered). **(c)** Breast invasive carcinoma (923 samples, 210 altered). **(d)** Glioblastoma multiforme (112 samples, 98 altered). **(e)** Uterine corpus endometrial carcinoma (mutation dominated 155 samples, 110 altered).

The most frequent common targets in the result groups are PIK3R1, HRAS, BRAF, MYC, RAC1 and RHOC. We detect mutually exclusive alterations at the upstream of RHOC in five datasets (Figure 4). RHOC is a member of Rho GTPases, whose members function in the regulation of cell shape, attachment and motility. Even though *RHOC* alterations are not frequent in TCGA samples, its over-expression was previously shown to promote metastasis of cancer cells [41,42]. We observe that it is expressed in a great majority of the TCGA samples (Additional file 1: Figure S46). These mutually exclusive alterations at the signaling upstream of RHOC in several different cancers suggest that activation of RHOC could be one of the major downstream effects of driver alterations.

### Comparison of methods that detect mutually exclusive gene alterations

We compared the performance of our method (Mutex) with the performance of previously published methods – Pairwise search [5], RME [6], Dendrix [8], MEMo [7], MDPFinder [9], Multi-dendrix [10] and ME [11] – for simulated datasets.

**Figure 5:**
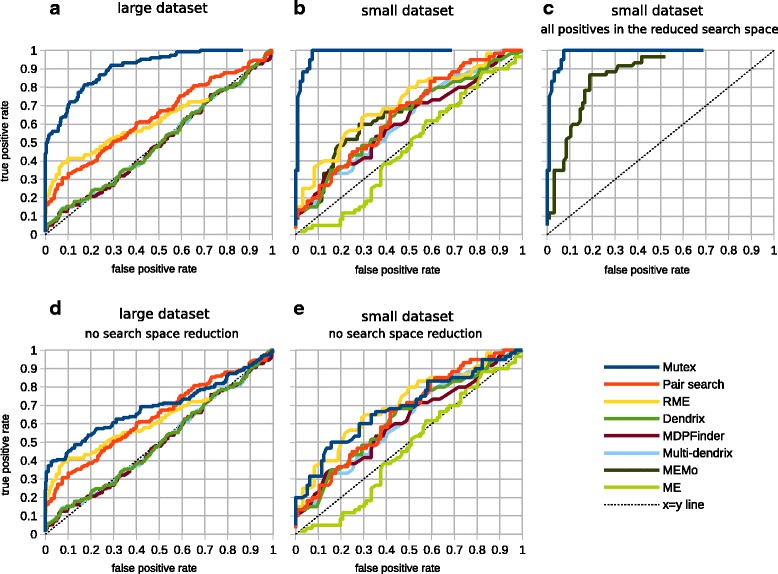
**Receiver operating characteristic curve comparisons of methods that detect mutually exclusive alterations for simulated datasets.** **(a)** Comparison with the large dataset. **(b)** Comparison with the small dataset. **(c)** Comparison of methods that do search space reduction (Mutex and MEMo) after ensuring all seeded groups are in the reduced search space of both methods. **(d, e)** Comparisons after removing the search space reduction of Mutex, for large and small datasets.

In our first trial, we derived a large dataset from the breast cancer dataset in cBioPortal (using mutations and expression-confirmed copy number changes), which contains 830 genes with an alteration rate of at least 3% in 958 samples. The derivation steps are: (i) randomize the sample distribution of gene alterations while preserving alteration ratios, (ii) determine 50 non-overlapping groups of genes, each with three members that are upstream of a common target in the signaling network, (iii) iterate over gene alterations in groups and re-randomize the overlapping alterations, and repeat once more. The last step decreases the chance of overlaps in the seeded groups. With this large dataset, we were not able to obtain results from MEMo and ME methods – neither algorithm scaled to the large data size. A comparison of receiver operating characteristic (ROC) curves (Figure 5a) shows a dramatic improvement over existing methods. However, note that this trial only captures the difference in performance for the groups of mutually exclusive genes whose members have a common downstream target in the signaling network. In a real case, there would be other groups missed by the gene network, but it is not easy to estimate their amount and to include these in a performance test.

Mutex outperforms the other methods for two reasons: (i) the reduction in search space and (ii) it has a more precise search metric. To understand how these two components contribute to the improvement, we compared a modified version of Mutex that does not use the signaling network and does not reduce the search space, with other methods that do not reduce the search space (Figure 5d). As expected, the ROC performance of Mutex decreased and became comparable with the other methods. The difference, however, is still substantial in terms of the number of results in a realistic use case. If we use a 5% FDR cutoff for the large dataset, modified Mutex recovers 49 true positive genes in the results while the next-best method, RME, recovers only 12.

This experiment validates that Mutex’s statistical metric is an improvement over other methods even without search space reduction. It also clearly demonstrates that exploiting the prior pathway information can improve precision. There is one caveat though – for real data, a common downstream molecule is one possible pattern out of many that can lead to mutually exclusive alteration patterns. The algorithm, however, is pattern agnostic and can be readily extended to other patterns – a research direction that we are exploring.

To include MEMo and ME in the comparison, we prepared a smaller simulated dataset (156 genes and 463 samples), and seeded 20 groups, each with three members, using the same procedure, and compared ROC curves (Figure 5b). Again, Mutex outperformed all other methods. In this comparison, most of the advantage of Mutex is from reducing the search space. MEMo is the only other method that reduces the search space. MEMo, however, has a disadvantage in this comparison because we did not select the seeded groups to appear also as nodes of a clique in the interaction network that MEMo uses. For a fair comparison, we added new interactions between seeded group members to MEMo’s interaction network database. This modification creates a clique for each seeded group that can be detected by MEMo. In this case (Figure 5c), the performance difference between Mutex and MEMo is mostly due to the more stringent metric used by Mutex, which ensures that each member contributes significantly to the group thus decreasing the number of false positives.

This evaluation indicates that among the published methods that do not use prior information, there were no performance improvement posterior to RME. Dendrix, MDPFinder and Multi-dendrix share the same weight function, and we previously noted its tendency to favor noise over signal. Confirming this behavior, for the large dataset we observe exceptionally poor performance (Figure 5a). ME uses a generative model for mutually exclusive alterations and scores the tested groups according to their fit to this model, compared to a random model. Their generative model assumes an equal chance for each gene in the group to be altered, so it only detects groups whose members have similar alteration ratios. Since we do not control for alteration ratios of genes in the seeded groups, ME performs poorly in our simulations (Figure 5b). We chose not to control it because there is not sufficient biological reason to expect genes in a group to have similar alteration ratios. We improve on both RME’s scoring criteria and MEMo’s search space reduction using gene networks.

The performance tests also demonstrate that Mutex scales well to large datasets and large groups, both in terms of memory usage and run time. It has similar run-time characteristics as RME and MDPFinder and is much more efficient than Dendrix, MEMo or ME (Additional file 1: Table S3).

## Discussion

Our approach detects many interesting genes and groups that are candidate cancer drivers and would not be detected by frequency-based methods such as MutSig. Additionally, mutually exclusive groups couple less known, less frequently altered genes with well-characterized cancer drivers, suggesting a mechanism of action. We also observe that many result groups are overlapping. We speculate that this suggests a highly coupled selection advantage between genomic modifications instead of well-defined modules or pathways.

Our mutual exclusivity score for groups of genes is analytical and fast to calculate for a single group. We still use permutation testing for multiple hypothesis correction when testing multiple groups. Since our method uses the same estimated null distribution for a gene regardless of the tested group, however, our approach scales substantially better compared to approaches where permutation testing is performed for each evaluated group.

We plan to extend this work towards searching other topological structures on the biological network. The current method selects genes with a common downstream target, and requires all group members to be directly linked on the network without a non-member linker node. Allowing linker nodes can help identify more distant mutual exclusion relations. The challenge of extending the search to other structures is that some structures can appear too many times in the network, expanding the search space and reducing the statistical power due to multiple hypothesis testing.

## Conclusions

We have developed a method that can detect mutually exclusive genomic alteration patterns in cancer genomic datasets. Mutex is unique in its ability to use prior pathway information efficiently to search the graph structure and reduce the search space. This reduction trades recall for precision. Given the highly noisy nature of the cancer genomic datasets, this is almost always a desirable trade-off. We also offer a new efficient and regularized statistical test that, even without the prior pathway information, improves the existing approaches.

## Materials and methods

### Mutual exclusivity of a pair

We define the alteration of two genes to be mutually exclusive if their overlap in samples is significantly less than expected by chance (Additional file 1: Figure S47). The statistical significance of the small overlap can be calculated using a hypergeometric test. For a pair of genes, a hypergeometric test is an analytical alternative to permutation testing, where permutations remove the dependency between alterations while preserving alteration rates, and assume equal probability that each sample is altered. This assumes a uniform alteration frequency from sample to sample. This might not always be the case, especially for the so-called hyper-mutated samples, which are often caused by a preceding mutation in DNA repair mechanisms. Properly addressing this heterogeneity is very complicated, as one should take into account that each overlap has a different probability in the null model. This is still an open problem. At the cost of statistical power, we partially mitigate this issue by excluding hyper-altered samples from the analysis. Specifically, we removed samples that have more alterations than Q3+(1.5×IQR) where Q3 is the third quartile in the distribution and IQR is the interquartile range. This criteria is often used in box plots to mark outlier values.

### Mutual exclusivity of a group

There is no standard way of testing whether a group of more than two genes exhibits a mutual exclusion pattern. We can compare pairs of genes, or we can get the union of alterations of a subset of genes in the group and compare it to another disjoint subset. There are combinatorially many ways to test the significance of the mutual exclusion in a group of genes. Among a wide number of possibilities, here we identify a subset of these tests to measure mutual exclusivity.

To ensure that every member of the group significantly contributes to the pattern, we use the following null hypothesis:

*H*_0_: *The specific member gene in the group is altered independently from the union of other alterations in the group.*

We test *H*_0_ for each member by evaluating the co-distribution of the gene with the union of other genes (Figure 2a) using a hypergeometric test. For a group of *n* genes, this method generates *nP* values, which are probabilities for the independent distribution of each member gene. Since we would like each member to contribute to the pattern, we use the *P* value for the least significant member as the initial score of the group. A closed-form expression for this metric is provided in Equation 1, where *g*_*i*_ is the alterations of the *i*th gene in the group, *g*_*n*−*i*_ is the merged alterations of group members excluding the *i*th gene, and *H* is the hypergeometric test that generates the *P* value of mutual exclusivity of two array of alterations:

(1) $$  \text{initial score} = \operatorname{Max}_{i} H(g_{i}, g_{n-i})  $$

Since we are testing more than one group, this initial (uncorrected) score is affected by multiple hypothesis testing. To correct for multiple hypothesis testing, we first estimate the null distribution of the initial *P* values of each gene, then calculate the significance of the observed initial *P* value for each member. Among this second set of *P* values, we select the least significant as the multiple hypothesis testing corrected final score (Figure 2a).

To estimate the null distribution of the initial *P* value for a gene, we sample it by permuting alterations of that gene, and searching for the group with the maximum uncorrected score using the same greedy search method. We only permute the alterations of the gene in question, because we are testing that gene in its specific network environment with a specific alteration pattern in surrounding genes.

To be able to control the FDR of the resulting groups, we need a measure for significance of the final score. Most popular FDR methods, like the Benjamini–Hochberg procedure, were developed for *P* values that are assumed to have a uniform null distribution. The final scores do not have a uniform null distribution even though they were derived from *P* values. This is due both to selecting the least significant *P* value in the group (which shifts the null distribution to the right) and searching for the best scoring set for a seed gene (which shifts the null distribution to the left). Since it is hard to estimate the shape of the null distribution analytically, we estimate it by running the analysis with all-permuted gene alterations, multiple times. Using the estimated null distribution of the final scores, we select the most significant results to satisfy a certain FDR. We also use this null distribution of final scores for estimating the value of (true positives − false positives), which we maximize while deciding a score cutoff.

### Verifying copy number alteration data with expression

To reduce the noise in the data, we filter out the copy number changes that are not supported by gene expression. We compare gene expression of copy number intact samples with samples that have the specific copy number alteration (either amplification or deletion) (Additional file 1: Figure S48). If the difference of mean expressions between two distributions is not in the expected direction or it is not statistically significantly different using a *t*-test with a 0.05 *P* value threshold, we do not use the copy number alterations in question. If the difference between the means is significant, we determine the expression threshold for which expression is more likely to belong to the copy number changed distribution, and use only the copy number changes with an expression satisfying the threshold. After this verification, if a gene still has both amplifications and deletions, we only use the type with the majority.

### Filtering genes

We filtered out genes that are not at the proximity of recurrently mutated or recurrently copy number altered genes in the signaling network. We used the MutSig analysis results with *q* value threshold 0.05 for recurrent mutations, and used Gistic results with *q* value threshold 0.05 for recurrent copy number alterations, both obtained through Broad Firehose. We define proximity genes as the union of the neighbor genes and the genes that have a common downstream target (the upstream of the downstream genes). We used the following procedure:

*A*= MutSig genes *B*= Gistic genes *C*= Proximity (*A*) *D*=*B*∩*CE*=*A*∪*DF*= Proximity (*E*)Result =*E*∪*F*

To improve performance, we filtered out genes with very low alteration rates. We used a minimum threshold of 0.01. The threshold was increased for studies that have very high overall alteration frequencies to ensure that the number of genes remains less than 500.

### Generation of simulated datasets

We derived the simulated datasets using the two TCGA breast cancer datasets deposited in cBioPortal. For the larger simulated dataset, we used the dataset named ‘Breast Invasive Carcinoma (TCGA, Provisional)’, which contained 958 complete samples at the time. Here, complete means that the sample has mutation profile, copy number profile and expression profile available. We used mutations and expression-verified copy number alterations as previously described. We filtered out genes with less than 3% alteration rate, which gave us 830 altered genes. For the small dataset, we used the dataset named ‘Breast Invasive Carcinoma (TCGA, Nature 2012)’, which contains 463 complete samples. We filtered out genes with less than 5% alteration, which gave us 158 altered genes.

We randomized the alterations of each gene separately, preserving the number of alterations, but re-assigning sample distributions. We chose 50 non-overlapping positive groups for the large dataset, and 20 positive groups for the small dataset, which are composed of three genes that have a common downstream gene in the signaling network. We applied the algorithm below twice to introduce artificially mutual exclusion to the alterations of positive groups by moving the overlapping alterations to new random locations:



### Software

An implementation of Mutex in Java is available online [45], distributed under GNU Lesser General Public License.

### Data availability

The codes of the TCGA datasets used in this manuscript are LAML, ACC, LGG, BRCA, COADREAD, GBM, HNSC, KIRC, KIRP, LUAD, LUSC, OV, PRAD, SKCM, STAD, THCA and UCEC. Processed versions of these datasets in the form of an alteration matrix are included in Additional file 2.

## Additional files

Additional file 1
**Supplementary text and figures.** PDF file containing graphical results of the analysis of each TCGA cancer dataset, and supplementary figures, tables and text.

Additional file 2
**Datasets and result details.** Zip archive with the datasets used in the study, the signaling network, result gene groups, significances, oncoprints, and other output result files. It also contains the simulated datasets and related networks.

## Abbreviations

FDR: False discovery rate
ROC: Receiver operating characteristic
TCGA: The Cancer Genome Atlas
