# Supplementary material for: Systematic identification of cancer driving signaling pathways based on mutual exclusivity of genomic alterations
Source: Genome Biol. 2015 Feb 26;16(1):45. doi: 10.1186/s13059-015-0612-6 (PMC4381444; doi:10.1186/s13059-015-0612-6)
Supplement: Additional file 1 — Supplementary text and figures. PDF file containing graphical results of the analysis of each TCGA cancer dataset, and supplementary figures, tables and text. [file 13059_2015_612_MOESM1_ESM.pdf]

Systematic identification of cancer driving signaling pathways based on mutual exclusivity

Özgün Babur, Mithat Gönen, Bülent Arman Aksoy, Niki Schultz,
Giovanni Ciriello, Chris Sander and Emek Demir

March 13, 2015

1 Results on TCGA cancer datasets

We analysed the mutual exclusivity of gene alterations for the following 17 TCGA studies:

- Acute Myeloid Leukemia (LAML)
- Adrenocortical Carcinoma (ACC)
- Brain Lower Grade Glioma (LGG)
- Breast Invasive Carcinoma (BRCA)
- Colorectal Adenocarcinoma (COADREAD)
- Glioblastoma Multiforme (GBM)
- Head and Neck Squamous Cell Carcinoma (HNSC)
- Kidney Renal Clear Cell Carcinoma (KIRC)
- Kidney Renal Papillary Cell Carcinoma (KIRP)
- Lung Adenocarcinoma (LUAD)
- Lung Squamous Cell Carcinoma (LUSC)
- Ovarian Serous Cystadenocarcinoma (OV)
- Prostate Adenocarcinoma (PRAD)
- Skin Cutaneous Melanoma (SKCM)
- Stomach Adenocarcinoma (STAD)
- Thyroid Carcinoma (THCA)
- Uterine Corpus Endometrial Carcinoma (UCEC)

4 of the datasets (Adrenocortical Carcinoma, Kidney Renal Clear Cell Carcinoma, Kidney Renal Papillary Cell Carcinoma, and Lung Squamous Cell Carcinoma) did not yield any result set with FDR smaller than 0.5.

The alterations in the endometrial cancer samples are either strongly dominated by copy number changes, or by mutations (Fig. S1). To eliminate the effect of these subtypes, we separated the dataset into two as CNA-domiated and mutation-dominated, and coded them with UCEC-cna and UCEC-mut, respectively.

We summarize the results of each TCGA study using 3 figures. The first figure is the plot of the expected number of true positivies and false positives in the results versus the false discovery rate (FDR) cutoff. Since we would like to maximize the true positives and minimize the false positives, we select the FDR cutoff that gives the maximum expected value of *true positives − false positives*. Second figure shows groups in the results. In these figures, the member genes of groups are nested in a compound node, and the border label of the compound node shows the sample coverage of alterations. When none of the common targets of the group is a member, one of the common

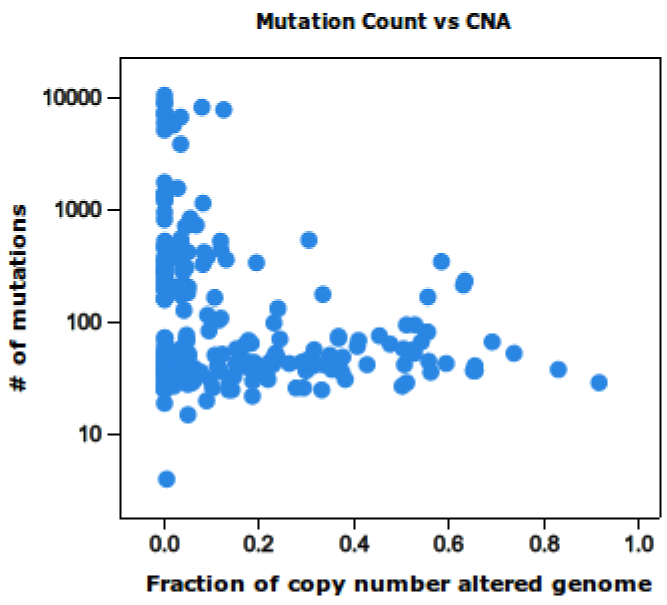

Figure S1: Distribution of the copy number alterations and mutations to endometrial cancer samples, as provided by cBioPortal.

targets are shown and placed outside of the compound node. Third figure shows the portion of the signaling network that contains the genes in the result groups and some of their common targets. In both of the second and the third figures, the gene colors are scaled to their alteration ratio. Table S1 shows the matching of the figures and the studies. Other details about the analyses – such as scores, p-values, oncoprints, etc – are given in a separate archive named "datasets-and-results.zip".

Table S1: The mapping between TCGA datasets and result figures.

Dataset name	FDR-guide	Result groups	Integrated network
Acute Myeloid Leukemia	Figure S2	Figure S3	Figure S4
Brain Lower Grade Glioma	Figure S5	Figure S6	Figure S7
Breast Invasive Carcinoma	Figure S8	Figure S9	Figure S10
Colorectal Adenocarcinoma	Figure S11	Figure S12	Figure S13
Glioblastoma Multiforme	Figure S14	Figure S15	Figure S16
Head and Neck Squamous Cell Carcinoma	Figure S17	Figure S18	Figure S19
Lung Adenocarcinoma	Figure S20	Figure S21	Figure S22
Ovarian Serous Cystadenocarcinoma	Figure S23	Figure S24	Figure S25
Prostate Adenocarcinoma	Figure S26	Figure S27	Figure S28
Skin Cutaneous Melanoma	Figure S29	Figure S30	Figure S31
Stomach Adenocarcinoma	Figure S32	Figure S33	Figure S34
Thyroid Carcinoma	Figure S35	Figure S36	Figure S37
Uterine Corpus Endometrial Carcinoma (CNA)	Figure S38	Figure S39	Figure S40
Uterine Corpus Endometrial Carcinoma (Mut)	Figure S41	Figure S42	Figure S43

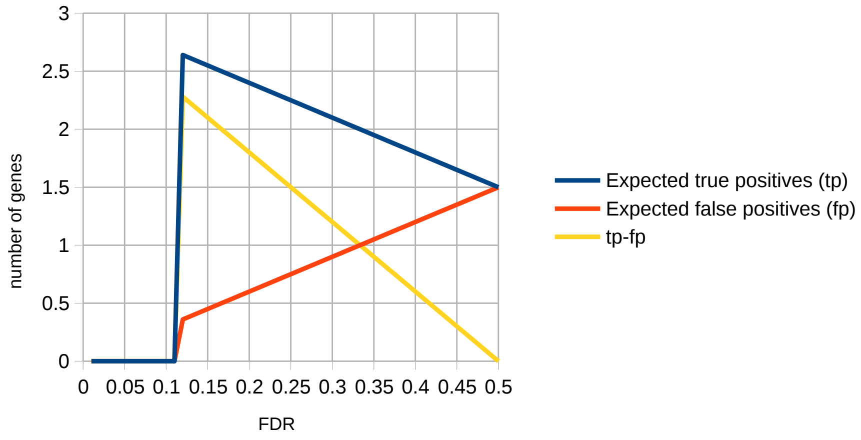

Figure S2: Change of expected number of true positives and false positives with FDR cutoff in Acute Myeloid Leukemia results.

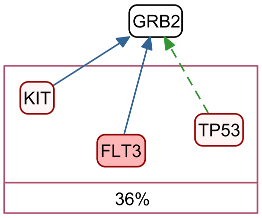

Figure S3: Groups of genes with mutually exclusive alterations for Acute Myeloid Leukemia.

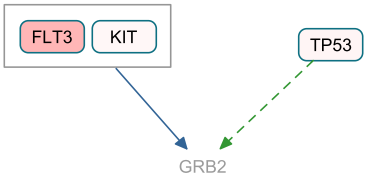

Figure S4: The signaling network identified using Acute Myeloid Leukemia analysis results.

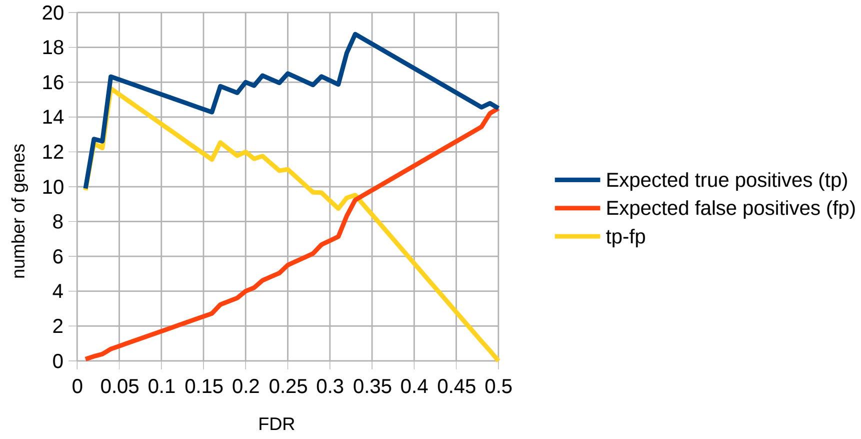

Figure S5: Change of expected number of true positives and false positives with FDR cutoff in Brain Lower Grade Glioma results.

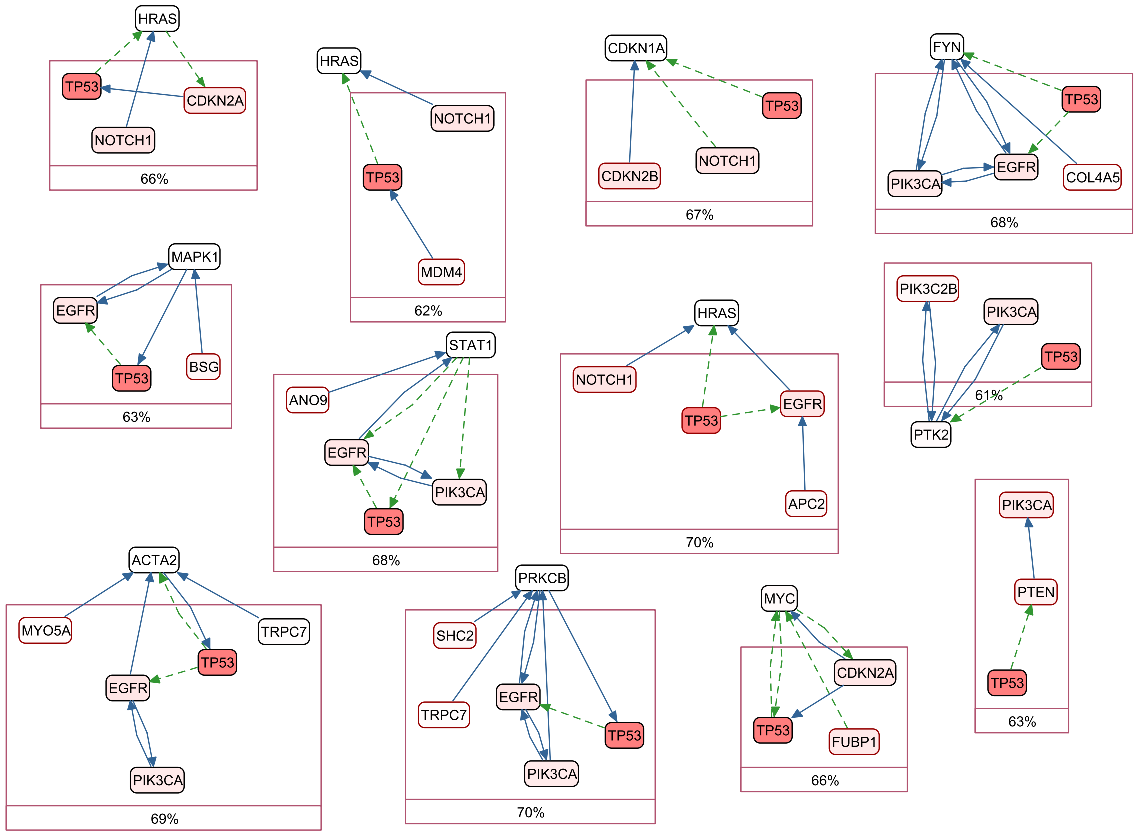

Figure S6: Groups of genes with mutually exclusive alterations for Brain Lower Grade Glioma.

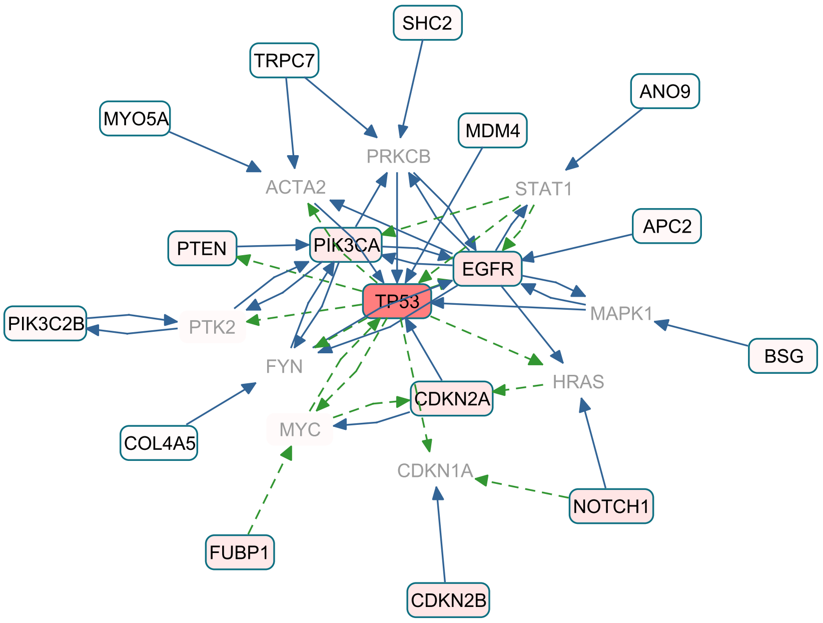

Figure S7: The signaling network identified using Brain Lower Grade Glioma analysis results.

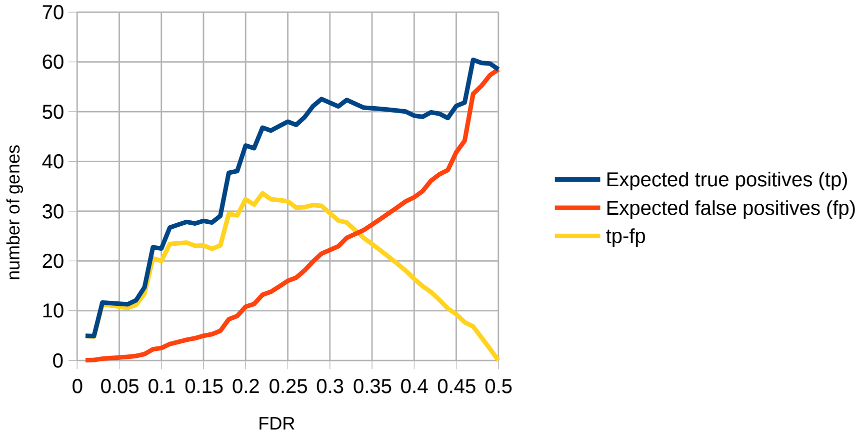

Figure S8: Change of expected number of true positives and false positives with FDR cutoff in Breast Invasive Carcinoma results.

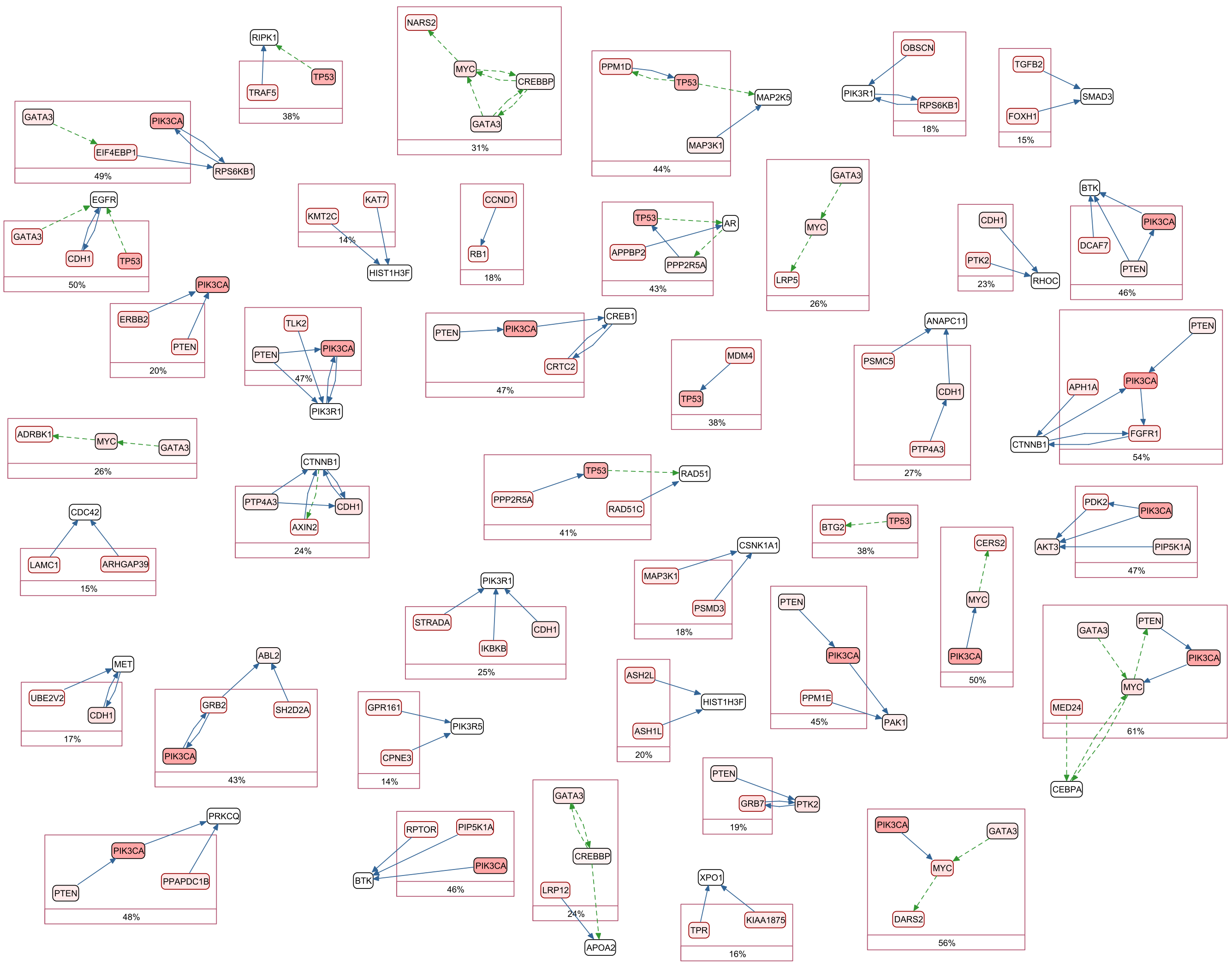

Figure S9: Groups of genes with mutually exclusive alterations for Breast Invasive Carcinoma.

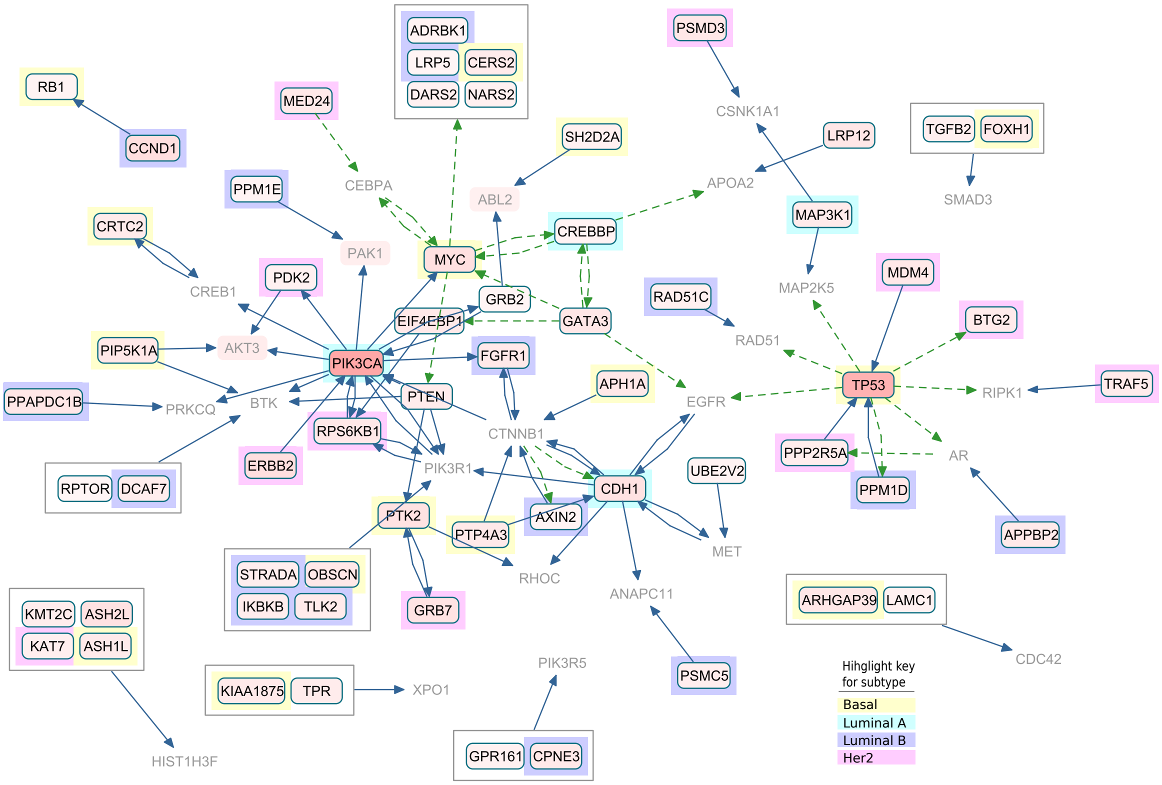

Figure S10: The signaling network identified using Breast Invasive Carcinoma analysis results. If a gene's alterations are significantly overlapping with a subtype, this is indicated with color-coded highlight.

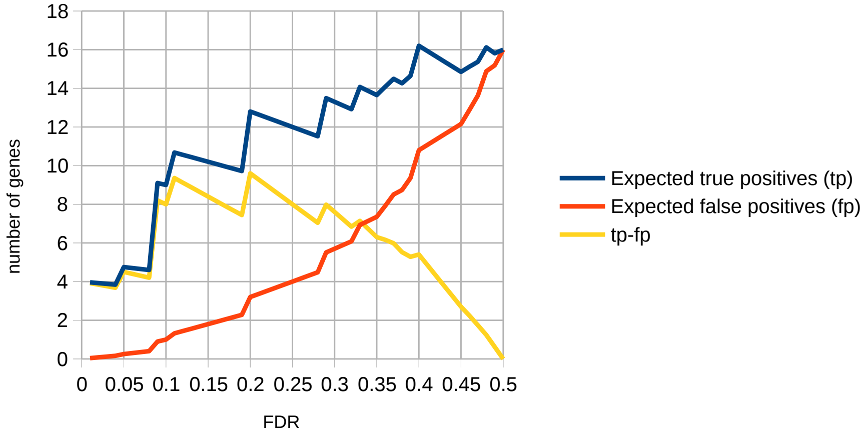

Figure S11: Change of expected number of true positives and false positives with FDR cutoff in Colorectal Adenocarcinoma results.

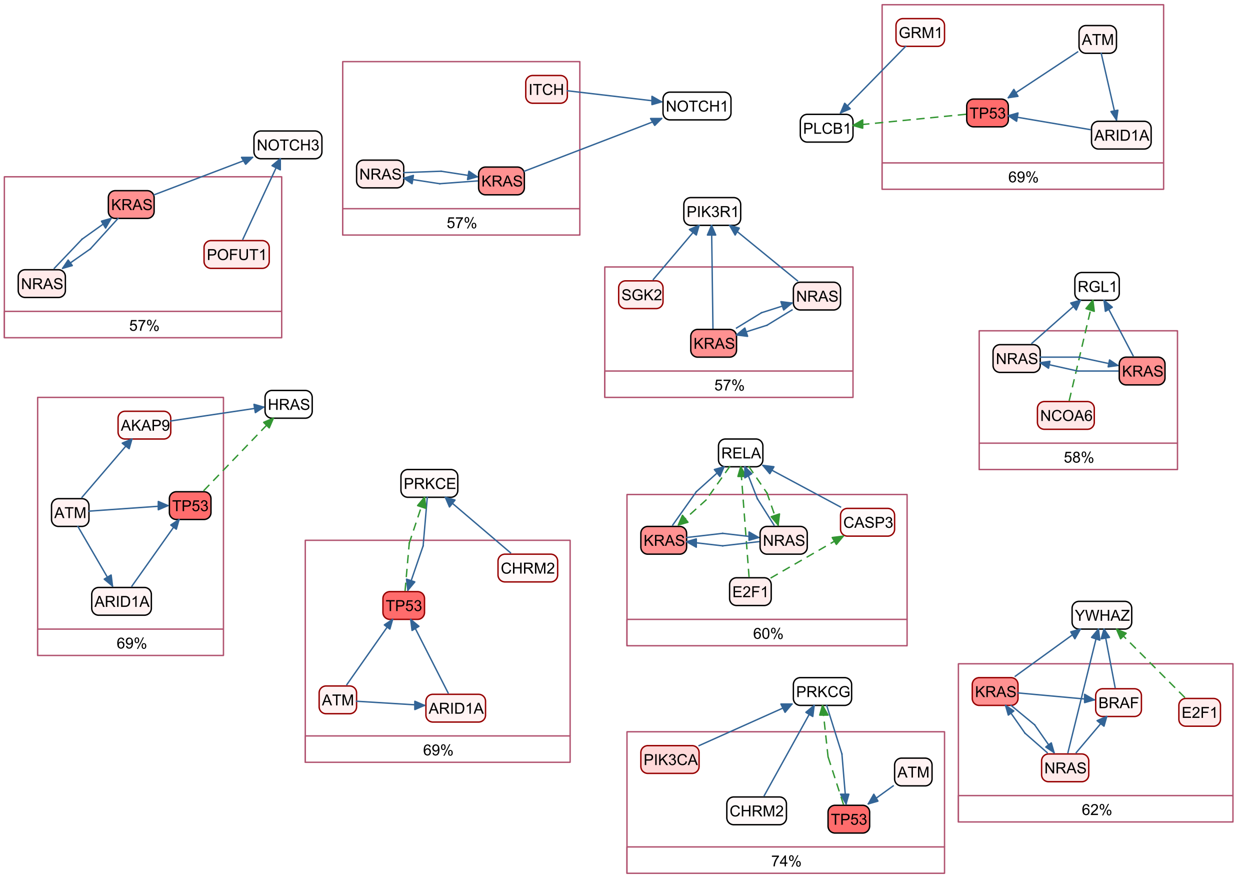

Figure S12: Groups of genes with mutually exclusive alterations for Colorectal Adenocarcinoma.

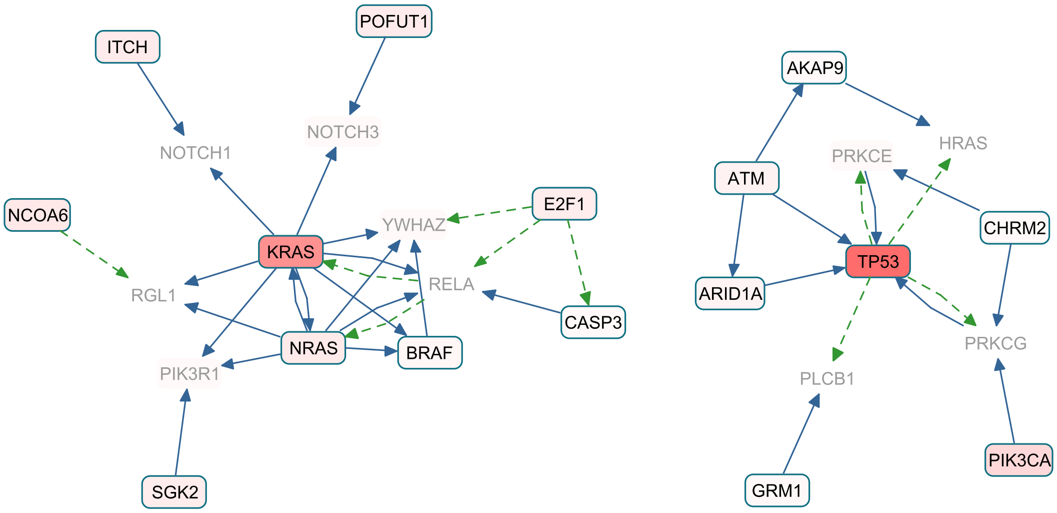

Figure S13: The signaling network identified using Colorectal Adenocarcinoma.

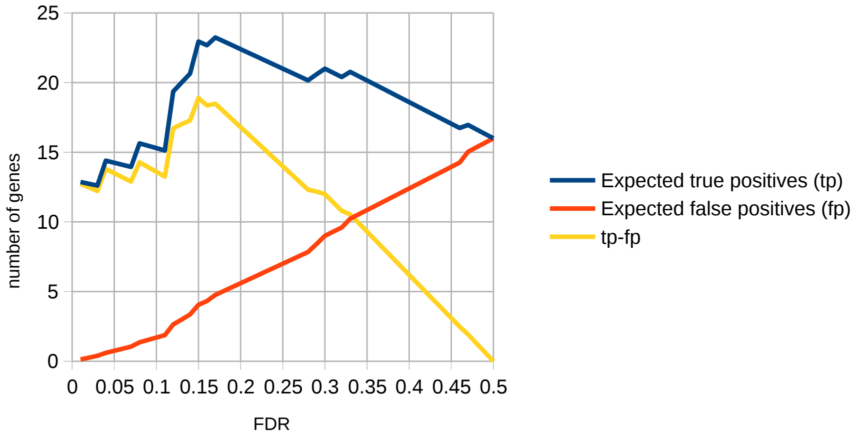

Figure S14: Change of expected number of true positives and false positives with FDR cutoff in Glioblastoma Multiforme results.

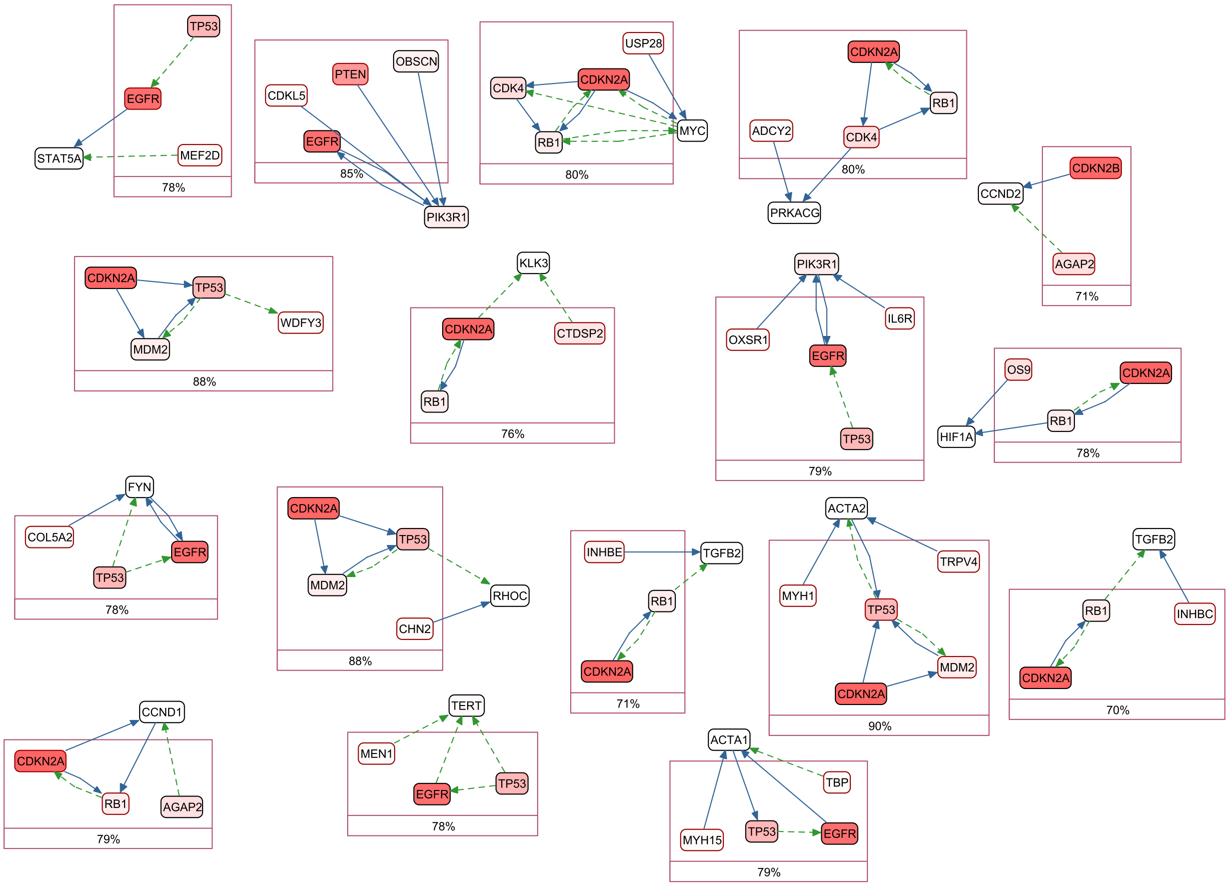

Figure S15: Groups of genes with mutually exclusive alterations for Glioblastoma Multiforme.

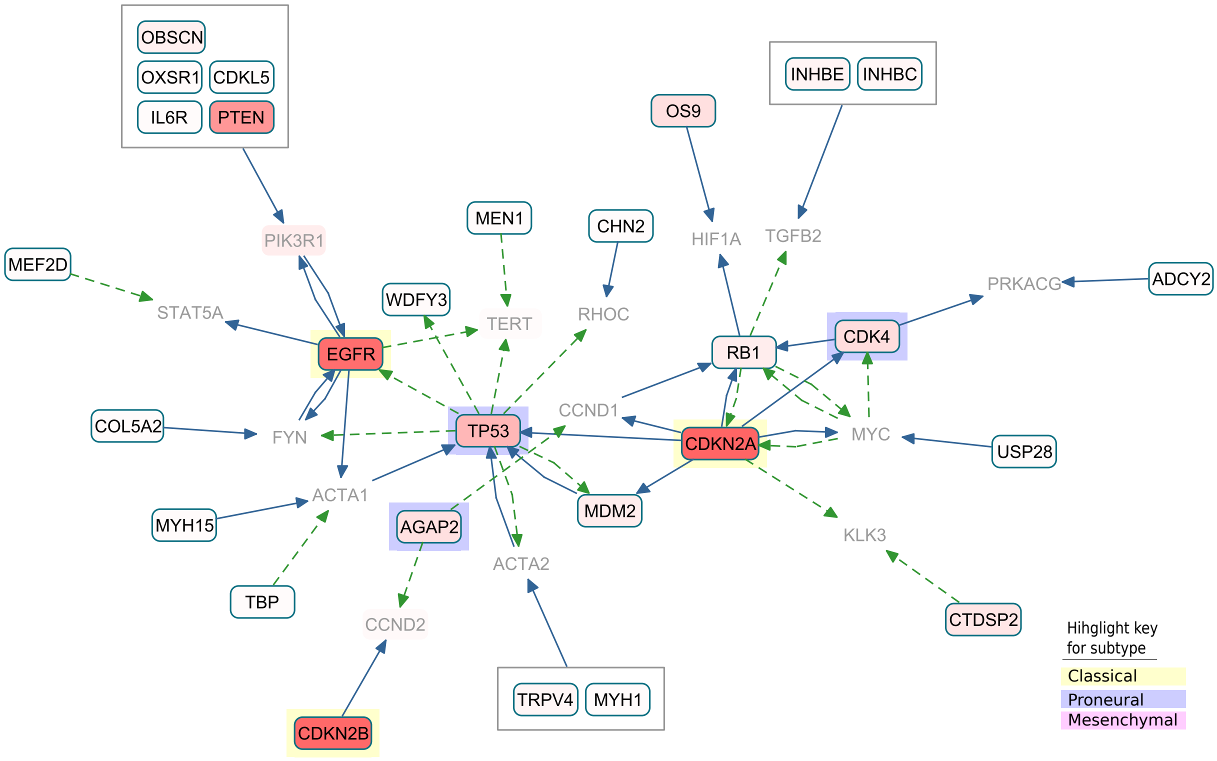

Figure S16: The signaling network identified using Glioblastoma Multiforme analysis results.

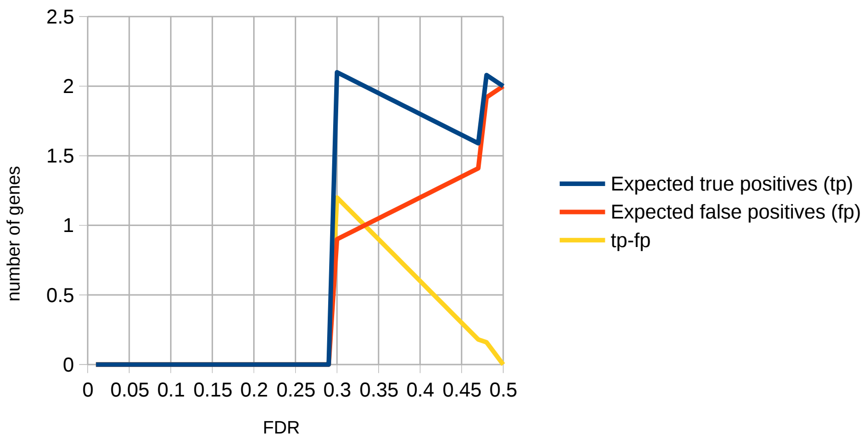

Figure S17: Change of expected number of true positives and false positives with FDR cutoff in Head and Neck Squamous Cell Carcinoma results.

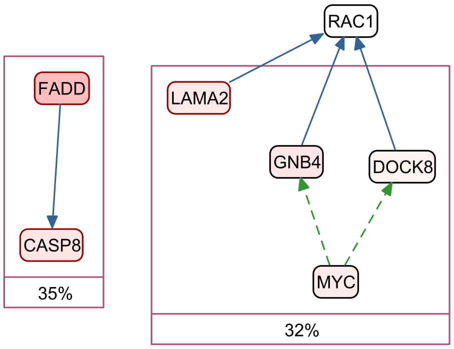

Figure S18: Groups of genes with mutually exclusive alterations for Head and Neck Squamous Cell Carcinoma.

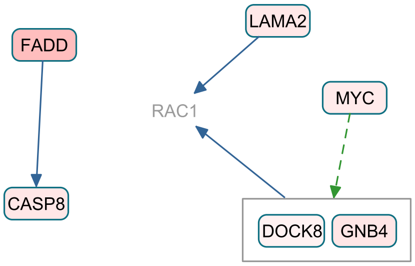

Figure S19: The signaling network identified using Head and Neck Squamous Cell Carcinoma analysis results.

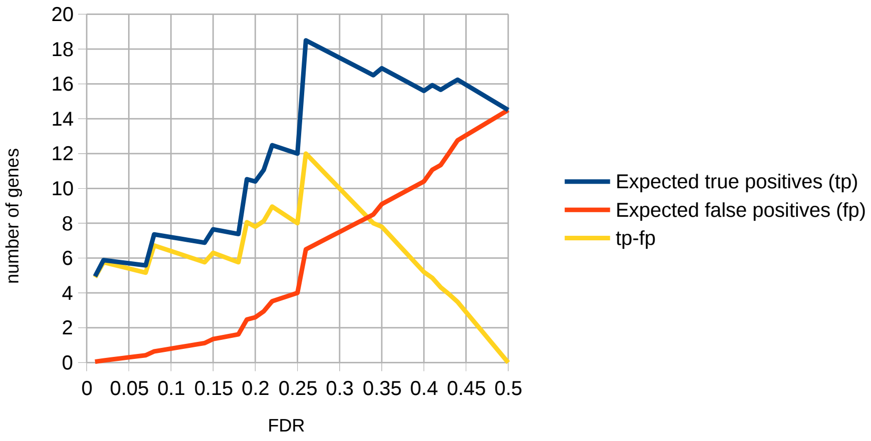

Figure S20: Change of expected number of true positives and false positives with FDR cutoff in Lung Adenocarcinoma results.

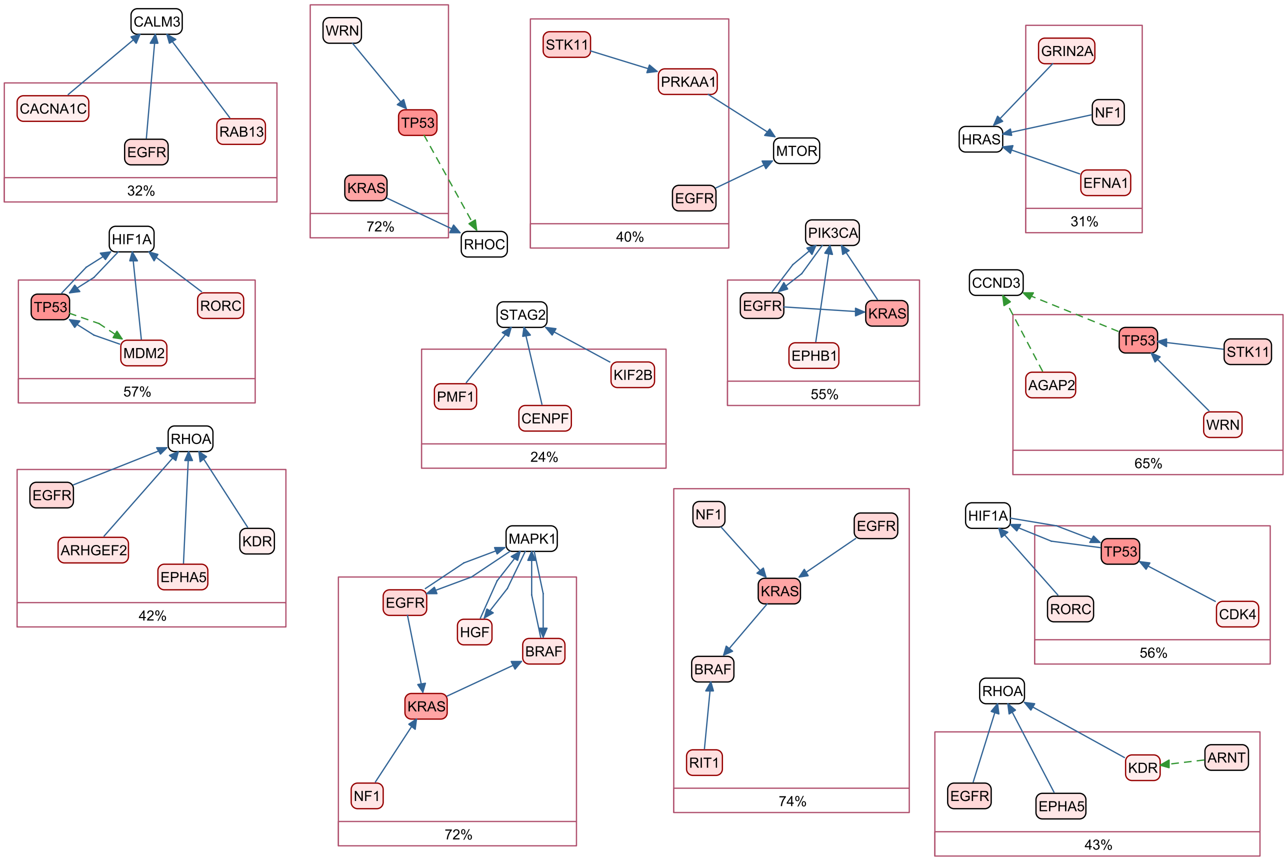

Figure S21: Groups of genes with mutually exclusive alterations for Lung Adenocarcinoma.

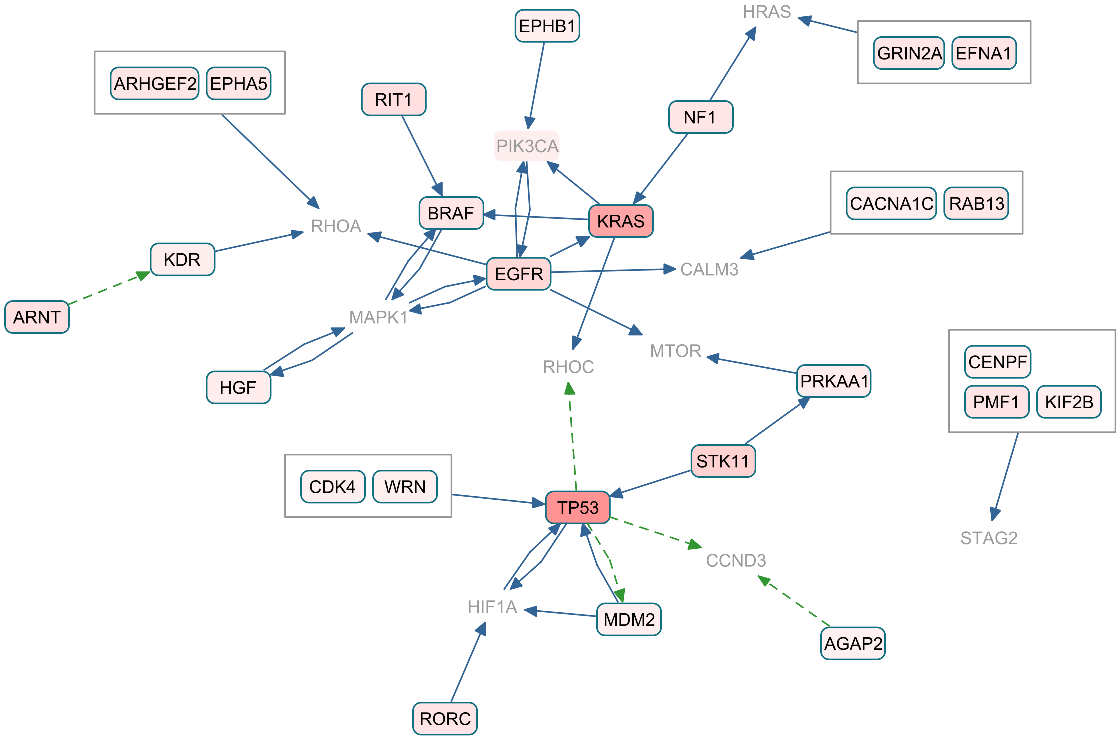

Figure S22: The signaling network identified using Lung Adenocarcinoma analysis results.

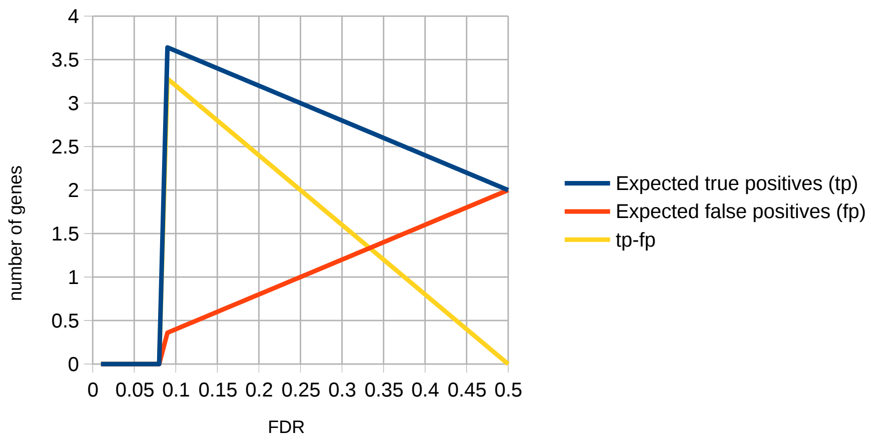

Figure S23: Change of expected number of true positives and false positives with FDR cutoff in Ovarian Serous Cystadenocarcinoma results.

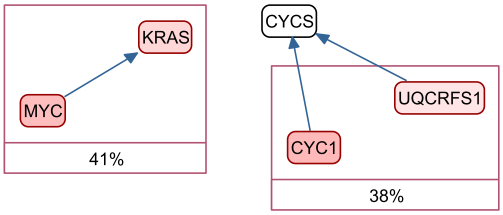

Figure S24: Groups of genes with mutually exclusive alterations for Ovarian Serous Cystadenocarcinoma.

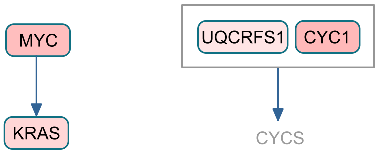

Figure S25: The signaling network identified using Ovarian Serous Cystadenocarcinoma analysis results.

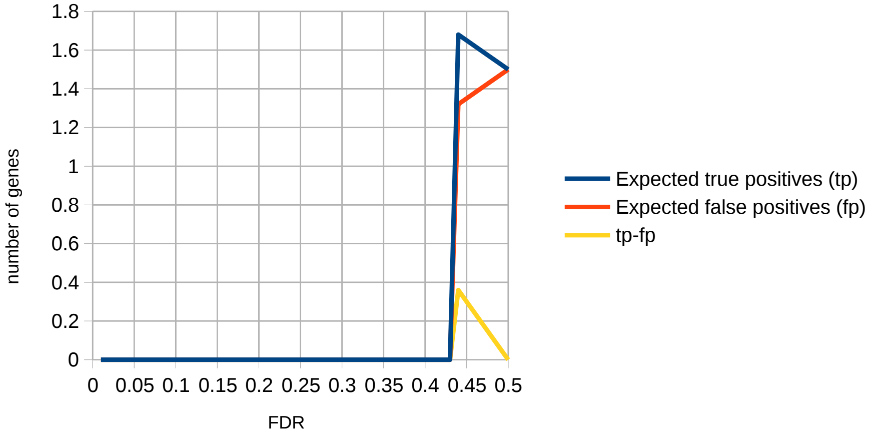

Figure S26: Change of expected number of true positives and false positives with FDR cutoff in Prostate Adenocarcinoma results.

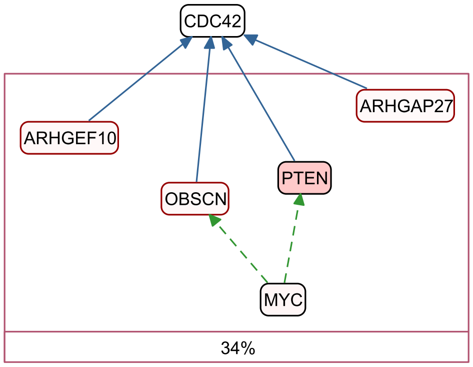

Figure S27: Groups of genes with mutually exclusive alterations for Prostate Adenocarcinoma.

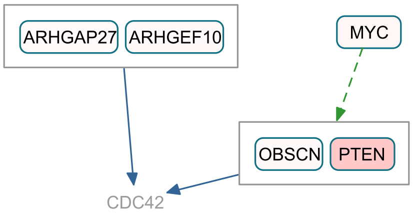

Figure S28: The signaling network identified using Prostate Adenocarcinoma analysis results.

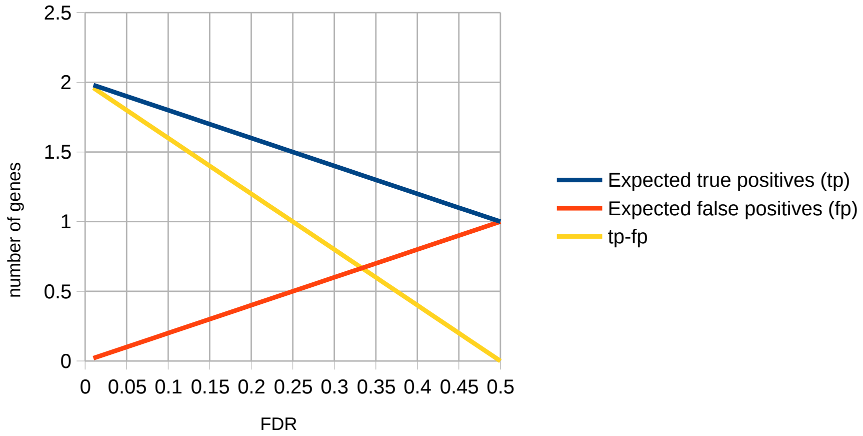

Figure S29: Change of expected number of true positives and false positives with FDR cutoff in Skin Cutaneous Melanoma results.

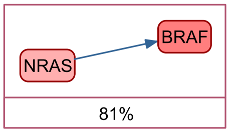

Figure S30: Groups of genes with mutually exclusive alterations for Skin Cutaneous Melanoma.

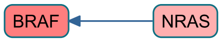

Figure S31: The signaling network identified using Skin Cutaneous Melanoma analysis results.

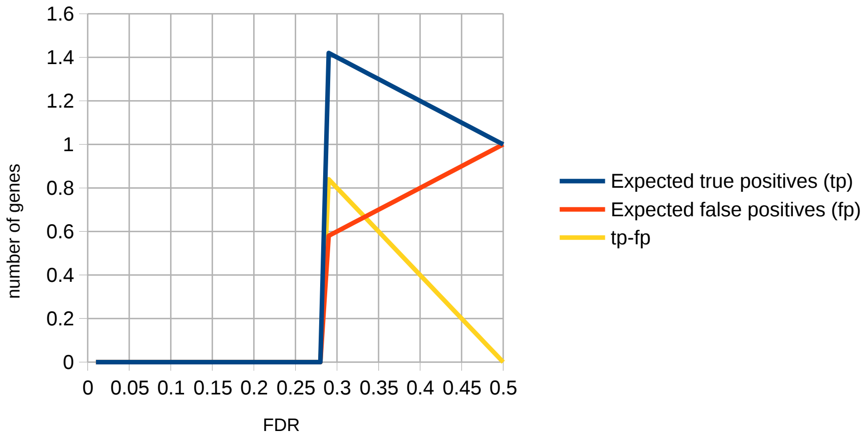

Figure S32: Change of expected number of true positives and false positives with FDR cutoff in Stomach Adenocarcinoma results.

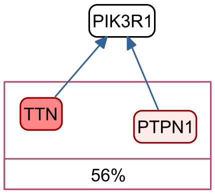

Figure S33: Groups of genes with mutually exclusive alterations for Stomach Adenocarcinoma.

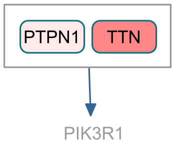

Figure S34: The signaling network identified using Stomach Adenocarcinoma analysis results.

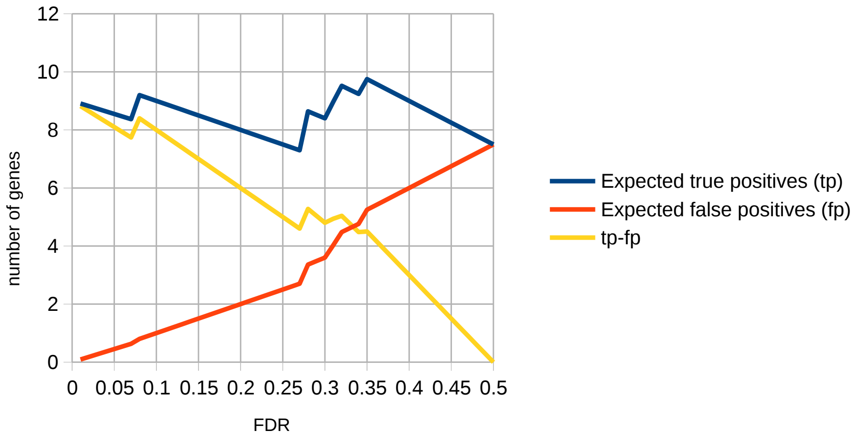

Figure S35: Change of expected number of true positives and false positives with FDR cutoff in Thyroid Carcinoma results.

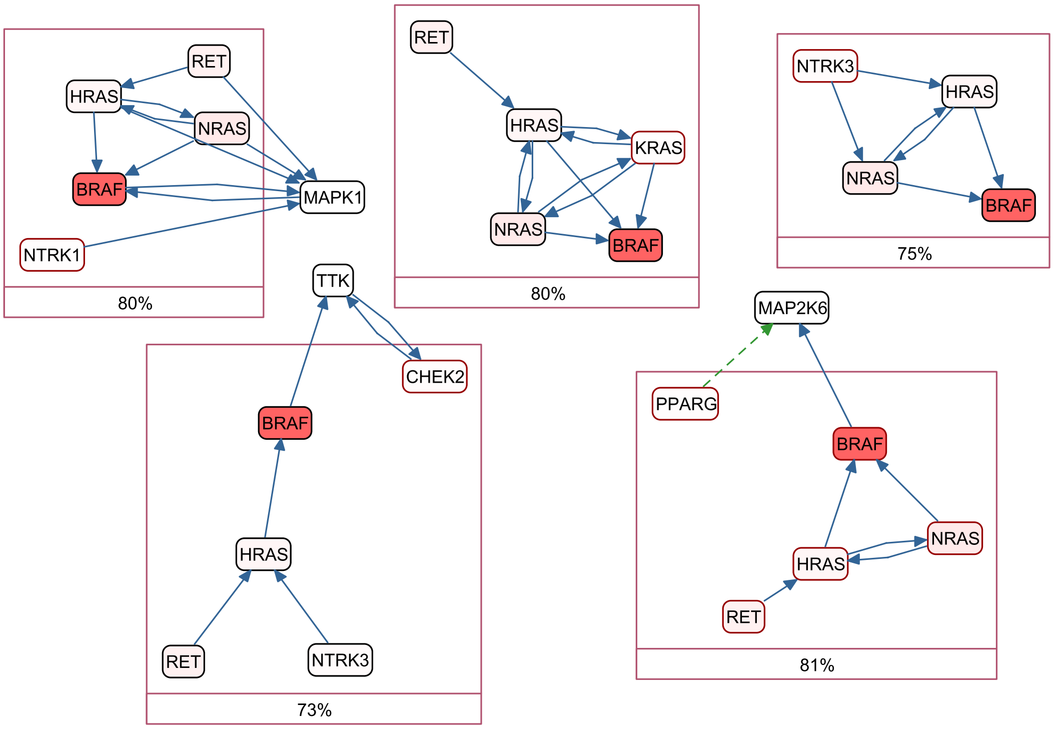

Figure S36: Groups of genes with mutually exclusive alterations for Thyroid Carcinoma.

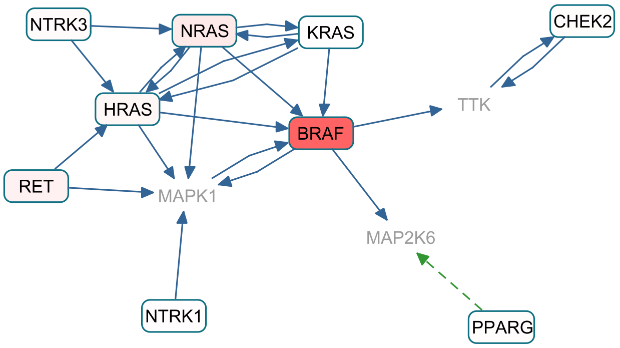

Figure S37: The signaling network identified using Thyroid Carcinoma analysis results.

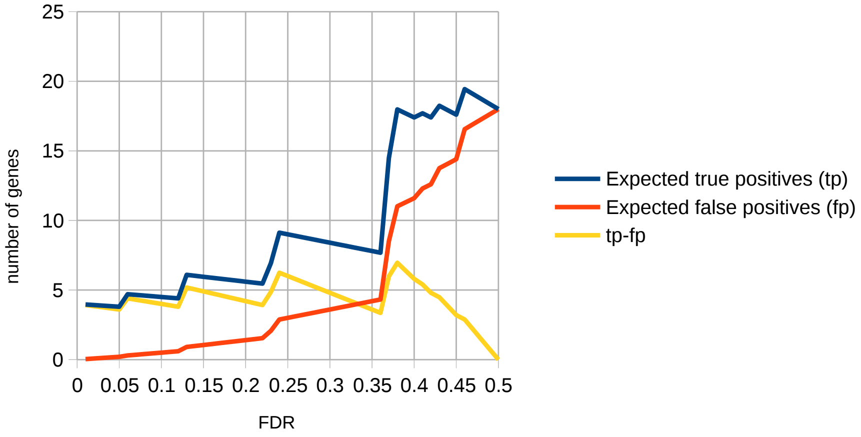

Figure S38: Change of expected number of true positives and false positives with FDR cutoff in the results of CNA-dominated samples of Uterine Corpus Endometrial Carcinoma.

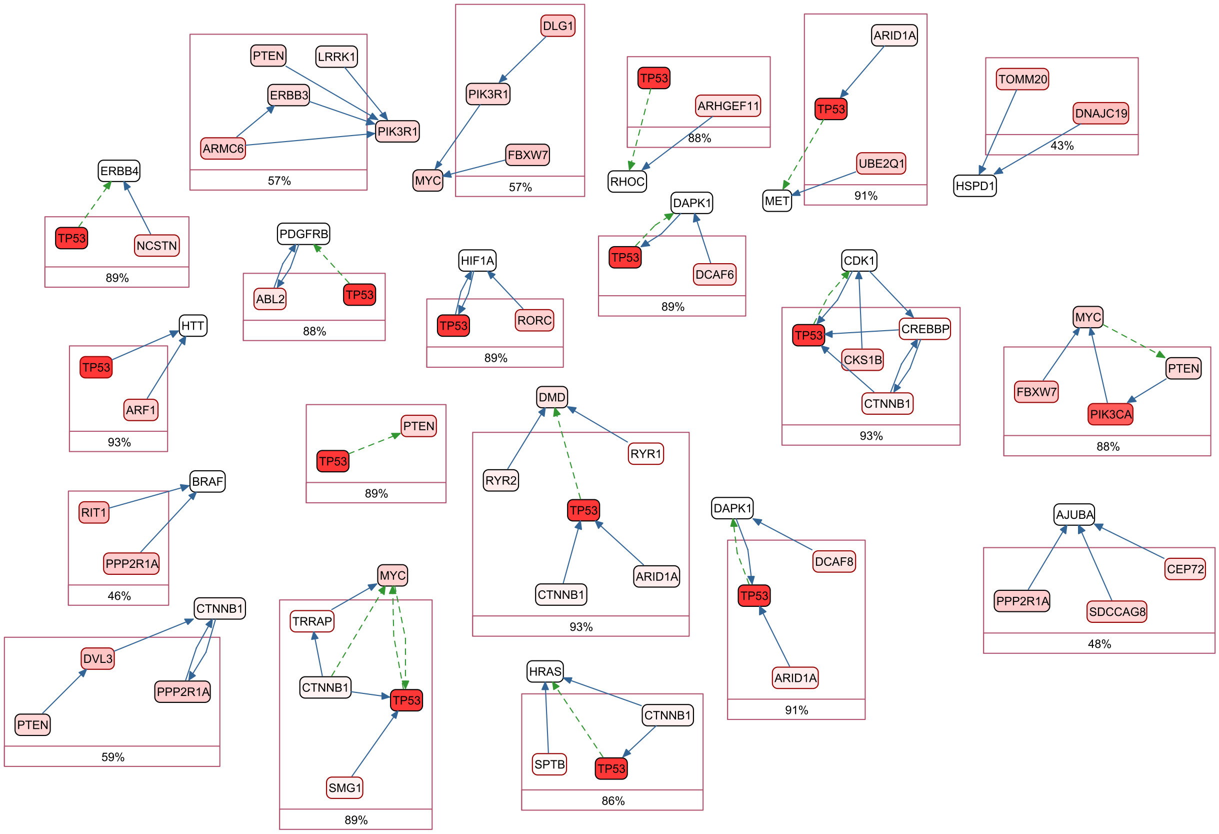

Figure S39: Groups of genes with mutually exclusive alterations for CNA-dominated samples of Uterine Corpus Endometrial Carcinoma.

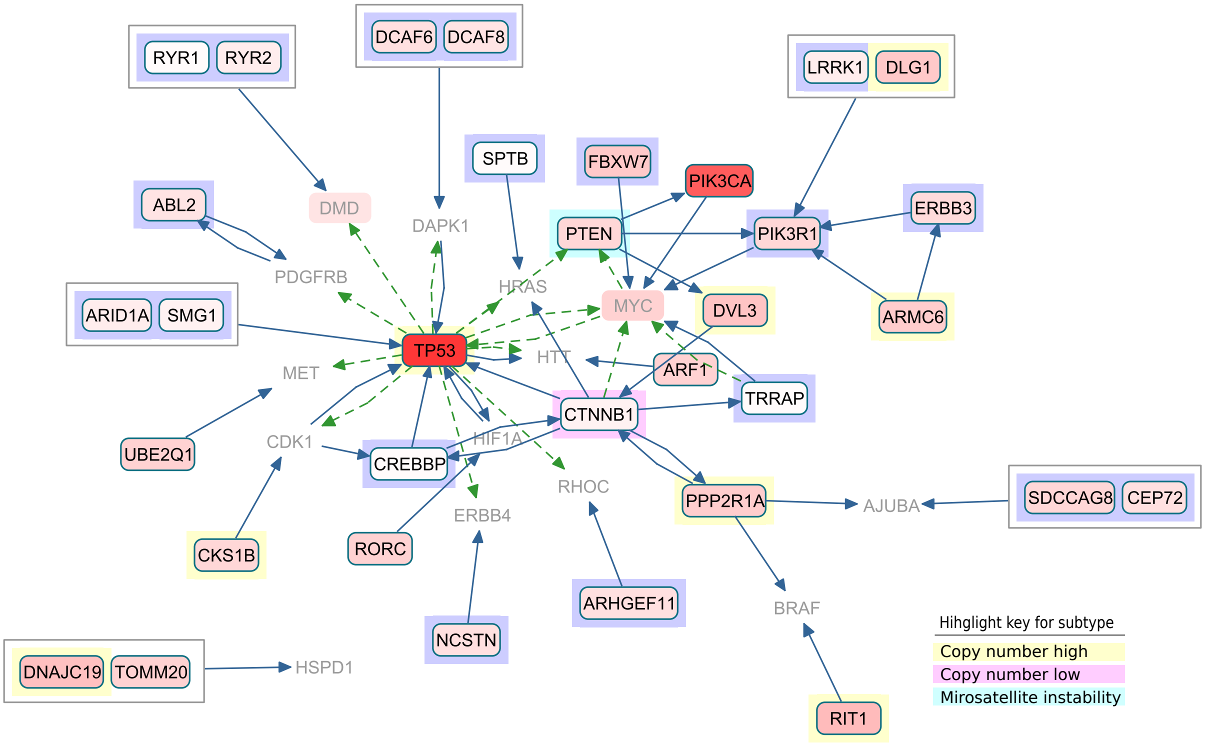

Figure S40: The signaling network identified using analysis results of CNA-dominated samples of Uterine Corpus Endometrial Carcinoma. If a gene's alterations are significantly overlapping with a subtype, this is indicated with color-coded highlight.

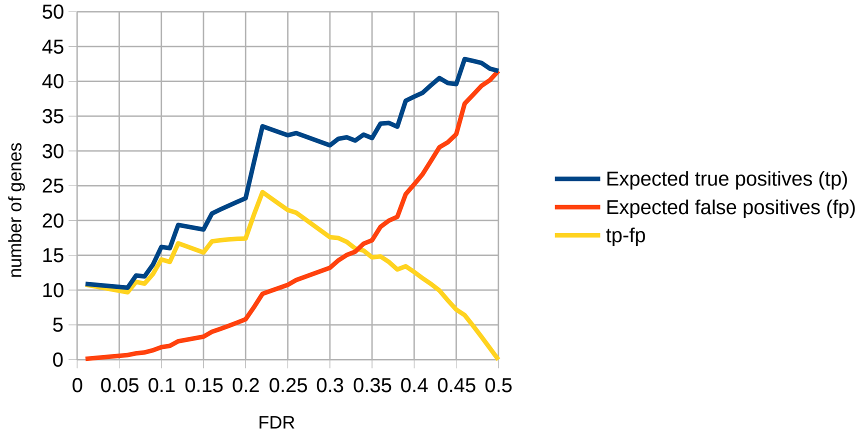

Figure S41: Change of expected number of true positives and false positives with FDR cutoff in the results of mutation-dominated samples of Uterine Corpus Endometrial Carcinoma.

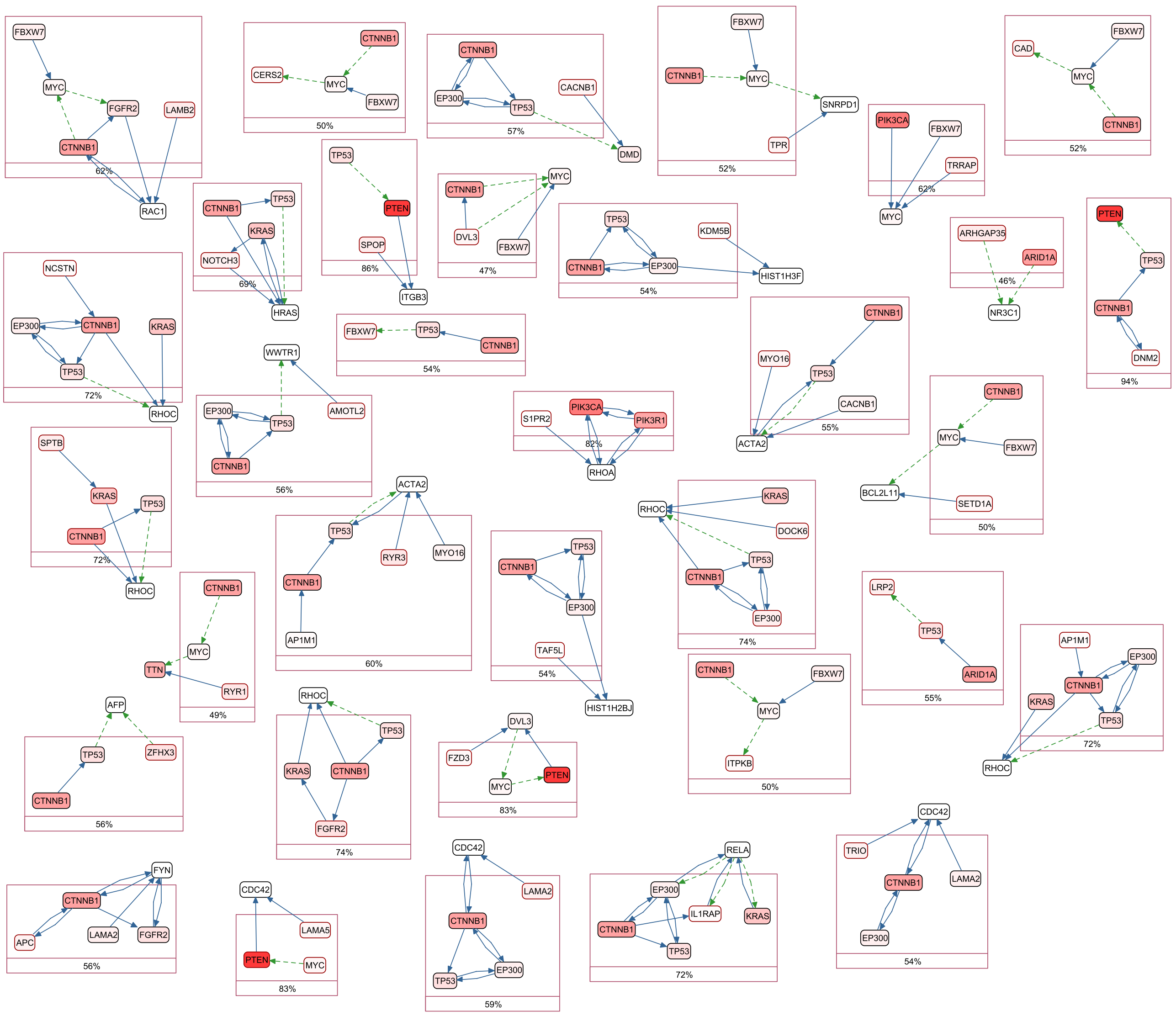

Figure S42: Groups of genes with mutually exclusive alterations for mutation-dominated samples of Uterine Corpus Endometrial Carcinoma.

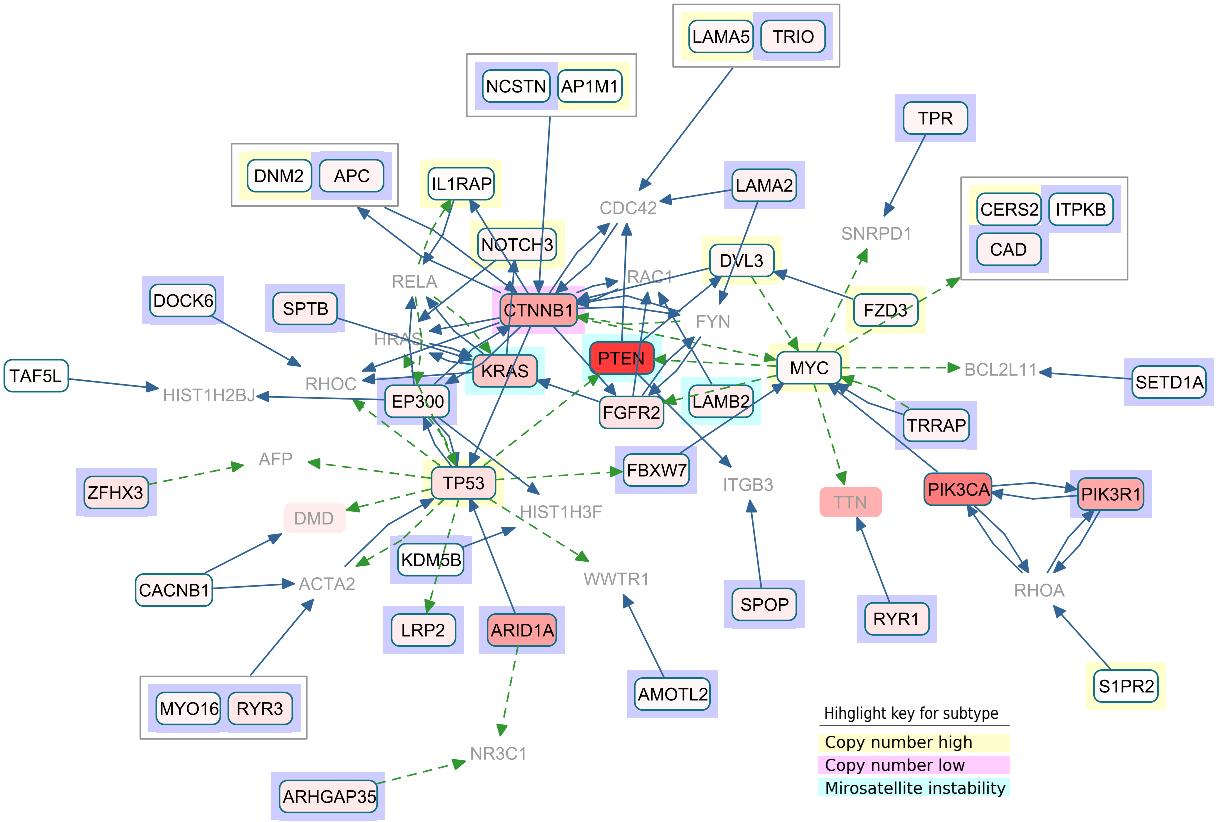

Figure S43: The signaling network identified using analysis results of mutation-dominated samples of Uterine Corpus Endometrial Carcinoma. If a gene's alterations are significantly overlapping with a subtype, this is indicated with color-coded highlight.

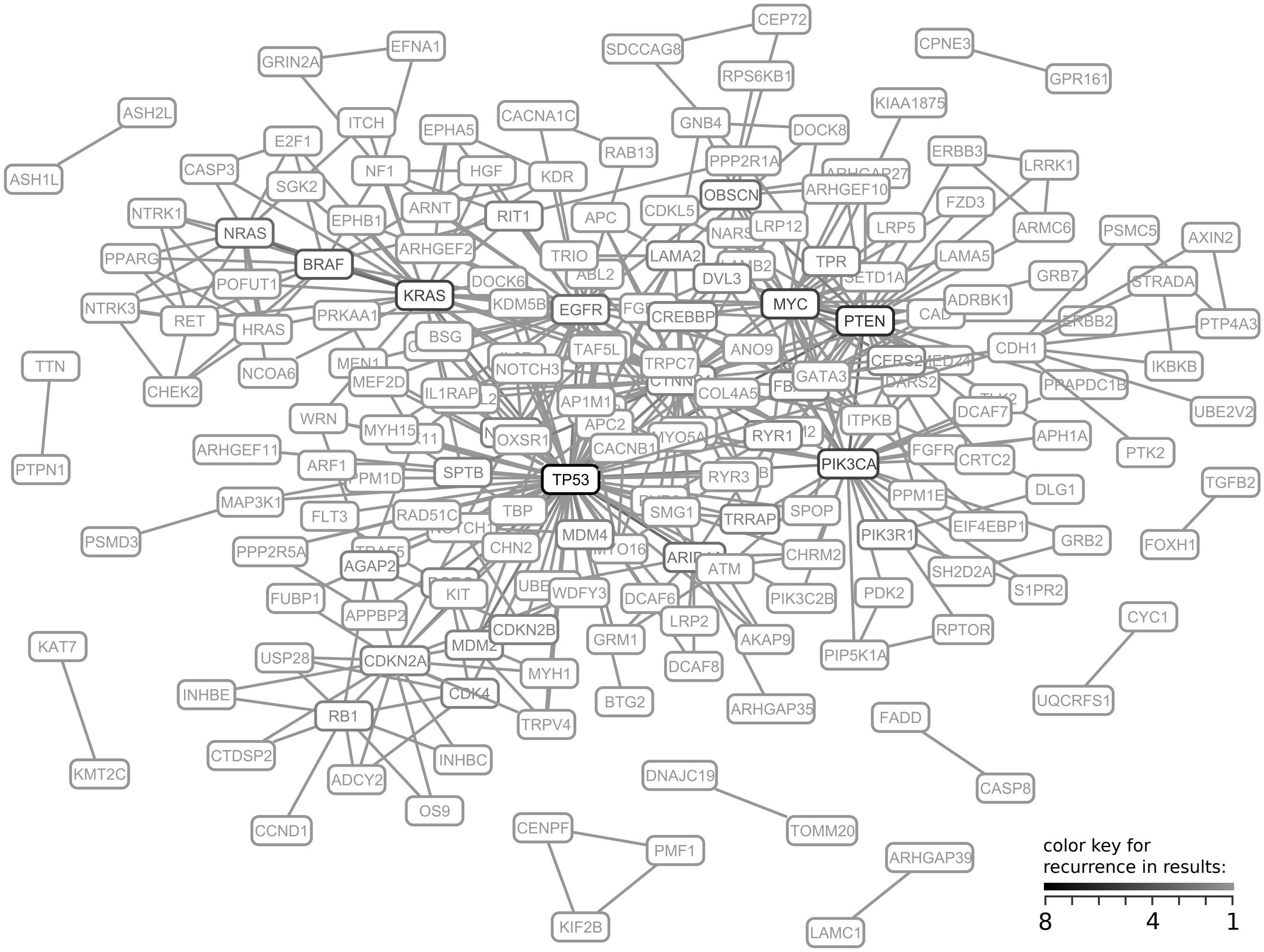

Figure S44: The complete version of Figure 3 of the main article.

Figure S44 is the complete version of Figure 3 in the main article. Here, recurrent and non-recurrent, all genes and co-presences in the results are shown. This graph contains 199 genes.

Table S2: 31 recurrent genes in results, sorted from well-known to least-known in the context of cancer. **Co-citation:** Co-citation count of the gene with the word "cancer" in PubMed articles [1]. **Mutex results:** Study codes that the gene is in Mutex results. If more than 3 studies exist, then only the number of studies displayed. If MutSig and Gistic cannot detect this gene in a particular study, that study is printed in bold color. **Recurrent mutations:** Study codes for which MutSig can detect this gene. **Recurrent copy number alterations:** Study codes for which Gistic can detect this gene. **Mutation hotspot:** Whether there is a hotspot in the overall distribution of mutations of the gene, among all the data submitted to cBioPortal.

Gene	Co-citation	Mutex results	Recurrent mutations (MutSig)	Recurrent copy number alterations (Gistic)	Mutation hotspot
TP53	5779	8 with **LAML**	13	GBM, PRAD	✓
EGFR	3984	GBM, LGG, LUAD	GBM, LGG, LUAD	5	✓
PTEN	2536	6	9	12	✓
KRAS	2441	5 with **THCA**	4	4	✓
CTNNB1	1957	UCEC-cna, UCEC-mut	ACC, PRAD, UCEC	KIRC	✓
TPR	1761	**BRCA, UCEC-mut**		COADREAD	✓
MYC	1707	5 with **HNSC**		8	✓
BRAF	1495	4	5	4	✓
MDM2	1408	GBM, LUAD		5	✓
CDKN2A	1033	GBM, LGG	4	11	✓
RB1	582	BRCA, GBM	5	12	✓
CDK4	549	GBM, LUAD		5	✓
PIK3CA	538	5	8	BRCA, KIRC, LGG	✓
CDKN2B	233	GBM, **LGG**		BRCA, GBM, KIRP	✓
FBXW7	138	UCEC-cna, UCEC-mut	4	KIRC	✓
MDM4	110	**BRCA**, LGG		COADREAD, GBM, LGG	
PIK3R1	90	UCEC-cna, UCEC-mut	4	BRCA, OV	✓
CREBBP	90	**BRCA**, UCEC-cna		LUSC, OV, UCEC	✓
NRAS	77	COADREAD, SKCM, THCA	4	6	✓
ARID1A	56	COADREAD, UCEC-cna, UCEC-mut	4	8	✓
DVL3	33	**UCEC-cna, UCEC-mut**		LGG	
TRRAP	21	**UCEC-cna, UCEC-mut**			✓
AGAP2	16	GBM, LUAD		5	✓
CERS2	15	**BRCA, UCEC-mut**		4	
RORC	11	**LUAD, UCEC-cna**		COADREAD, LUSC	✓
NCSTN	10	**UCEC-cna, UCEC-mut**		COADREAD	
LAMA2	5	**HNSC, UCEC-mut**		4	
RIT1	5	LUAD, UCEC-cna	LUAD	COADREAD, UCEC	✓
OBSCN	4	**BRCA, GBM**, PRAD	ACC	KIRC, PRAD, UCEC	✓
RYR1	3	**UCEC-cna, UCEC-mut**		LUSC	
SPTB	1	**UCEC-cna, UCEC-mut**		5	

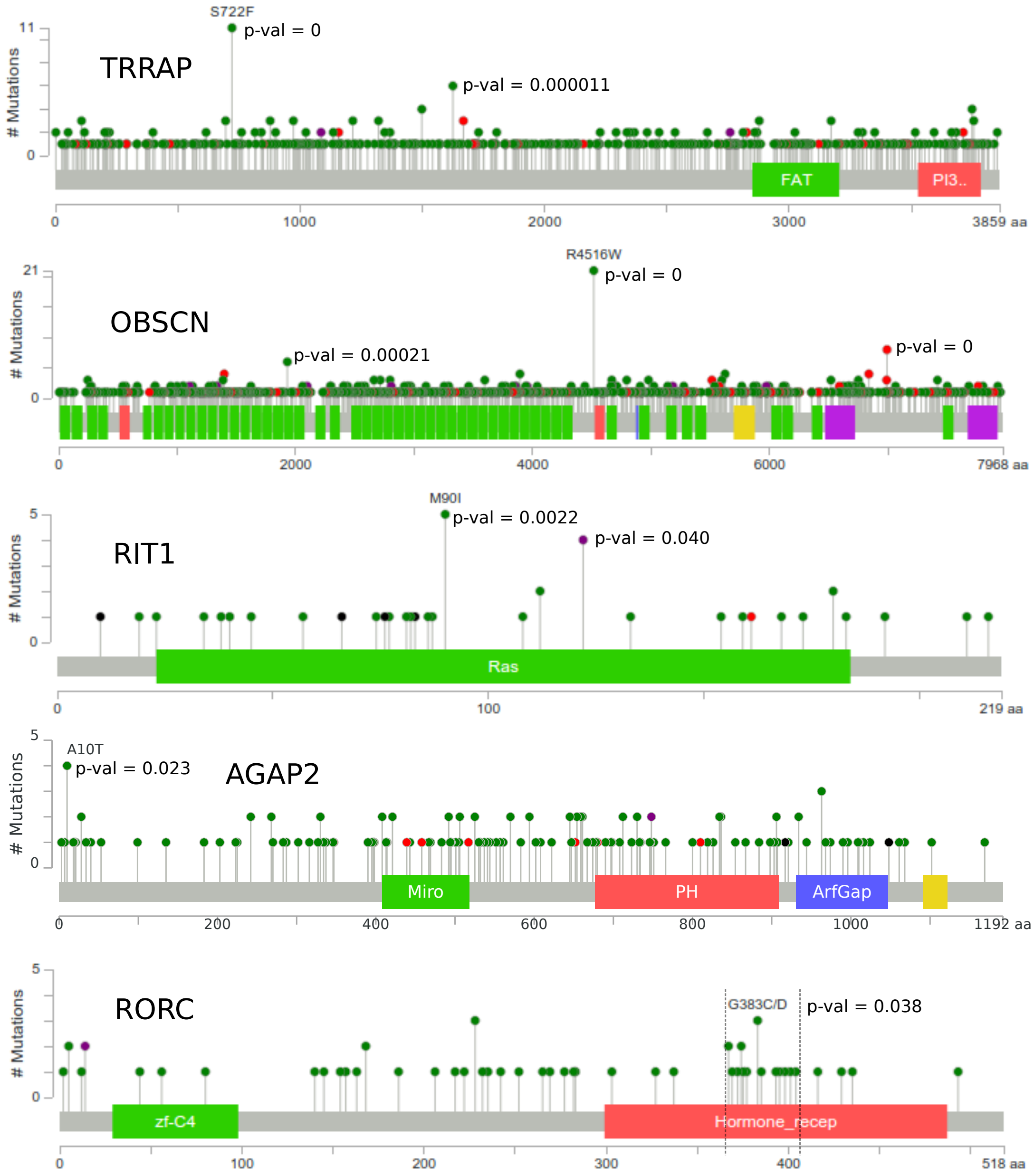

Figure S45: **Hotspots in the recurrent genes that are not strongly associated with cancer in the literature, as provided by cBioPortal.** Following is the key for mutation colors. Green: missense, red: either frameshift, or splice site, or nonsense, black: insertion or deletion, purple: mix of previous categories. Conserved protein domains are also shown on the graph with colored rectangles, where repeated domains have the same color. We tested significance of hotspots (and hot-region in the case of RORC), on 10 genes that are least-associated with cancer, by randomizing positions of the mutations 10^8 times. We found that the hotspots in 5 genes are statistically significant. The Benjamini-Hochberg procedure estimates 0.076 false discovery rate for these 5 genes. All the most significant hotspots on these genes are composed of missense mutations. We also detected a very significant hotspot on RYR1, but they are all frameshift mutations and most of them have very low allele frequencies, so we didn't include it in this figure.

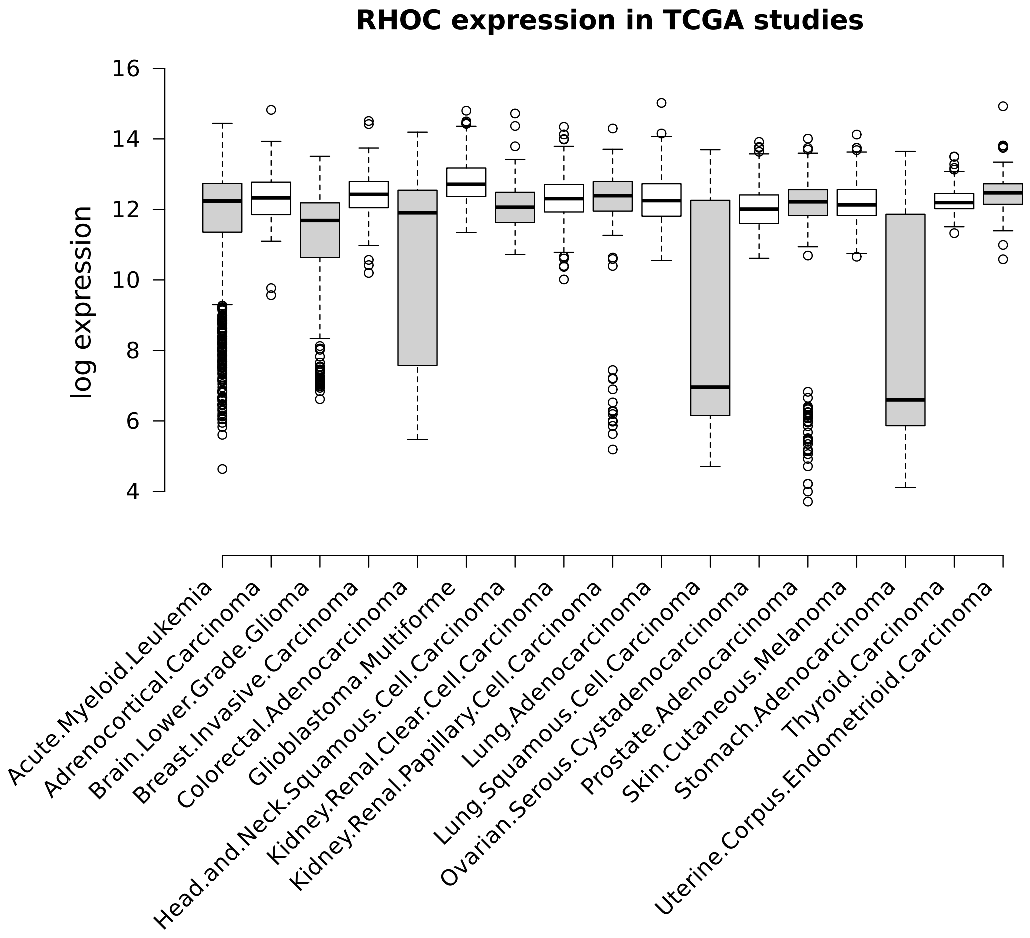

Figure S46: The distribution of RHOC expression in TCGA studies.

The recurrent target gene RHOC is expressed in majority of TCGA samples (Fig. S46).

2 Method runtimes in simulation

Table S3: The recorded runtimes of the compared methods in the simulation study.

Method	Large dataset	Small dataset	Large dataset (using network)	Small dataset (using network)
Mutex	13h, 6m, 42s	11m, 54s	1h, 5m, 55s	8m, 34s
Pair search	2s	less than a second	-	-
RME	11h, 52m, 36s	29m, 17s	-	-
Dendrix	1d, 21h, 45m, 40s	4h, 5m, 18s	-	-
MDPFinder	14h, 35m, 51s	1m, 12s	-	-
Multi-dendrix	35m, 41s	1m, 1s	-	-
MEMo	-	-	did not finish	9m, 58s
ME	did not finish	1h, 37m, 12s	-	-

3 Method figures

Figure S47 shows the mapping between the oncoprint notation and contingency table notation, both represent distribution of alterations in two genes. Figure S48 provides an example distribution where copy number amplified genes have higher expression.

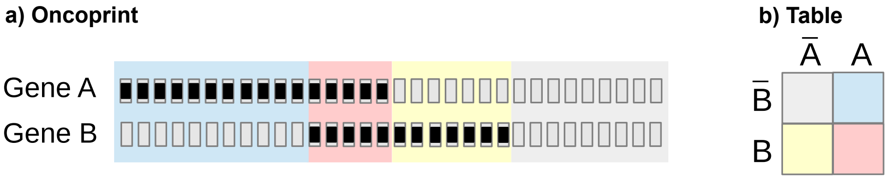

Figure S47: **Two different representations for the overlap in gene alterations of two genes.** Background colors marks and represent the sample counts for each four categories. a) Oncoprint notation. Each column of gray rectangles indicates a sample, and gene alterations are marked with black. b) A 2-by-2 contingency table showing counts of samples classified into 4. Hypergeometric test is commonly used to calculate p-value for dependency of attributes in such a table.

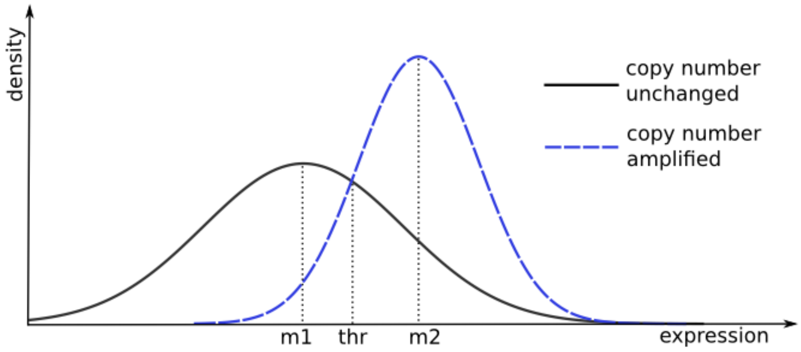

Figure S48: **Verification of copy number changes using expression data.** We only used copy number changes that are verified with expression. *m1* and *m2* are mean expressions of copy number intact and copy number amplified samples, respectively. *thr* is the threshold expression marking the point where an expression has higher probability to belong to the amplified samples.

4 Issues in methods that detect group mutexness

4.1 Cliques of pairwise mutexness

Yeang *et al.* [2] detect mutex gene pairs, and detect a group only if all pairs in the group are significantly mutex. This method is too strict to search for mutex groups and will fail to detect most groups since a significant mutex group typically contains some insignificant pairs. Figure S49 shows such a mutex group that cannot be detected by evaluating pairwise relations.

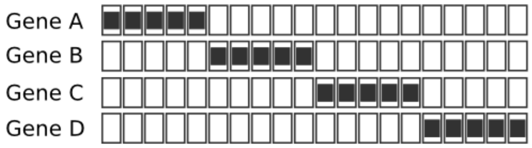

Figure S49: **Example of an extremely significant mutex group where each member contributes equally and mutexness of gene pairs are insignificant.** The chance to get a perfect mutex pattern with random permutation is 0.19 for any two genes, while it is 2×10^{-7} with all four genes.

4.2 Using coverage and overlap without considering significance

Vandin *et al.* [3], Zhao *et al.* [4] and Leiserson *et al.* [5] define the weight of a mutex group as "coverage - overlap", where coverage is the number of samples where at least one gene is altered, and overlap is the surplus of alterations over the coverage (Eq. 1-3).

$$\text{weight} = \text{coverage} - \text{overlap} \quad (1)$$
$$\text{overlap} = \text{total alteration count} - \text{coverage} \quad (2)$$
$$\text{weight} = (2 * \text{coverage}) - \text{total alteration count} \quad (3)$$

They argue that this weight function is a trade-off between coverage and overlap. However, since the significance of mutexness is not controlled, this measure is prone to detect noise in the system instead of the signal. Such an example is given in Figure S50, where the weight function will favor the second group (high weight and low significance) over the first group (low weight and high significance).

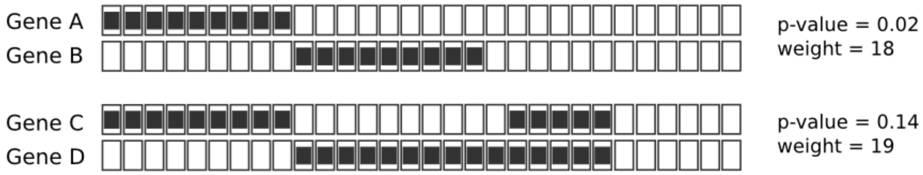

Figure S50: **Two example gene groups A-B and C-D, where mutexness of A-B is much more significant than C-D according to hypergeometric test, but group C-D has higher weight according to Eq. 3.**

The second drawback of this approach is that it fails to detect cases such as a gene in the group decreases the group weight while it is significantly mutex with the group. For instance the weight of gene A in Figure S51 is higher than the group A-B. This is because addition of gene B increases coverage by 5, but also increases overlap by 6, which results in a decrease in weight. In that case, the search function would detect gene A as a group of 1 rather than detecting the group A-B.

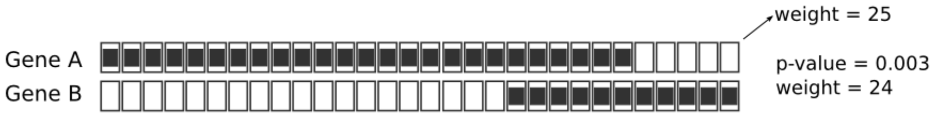

Figure S51: **A highly significant mutex group A-B, where the weight of gene A itself is higher than the group.**

5 Test for a degree-bias in Mutex's metric

During the peer-review of this manuscript, one of the reviewers noted the high frequency of the appearance of *TP53* in Mutex results, and asked if this can be due to the very high degree of *TP53* on the signaling network. To test if there is a degree-bias in our method, we calculated the spearman correlation between the rank of false genes in the simulation results of large simulated dataset, and their degree (number of neighbors) on the network. We expect this correlation to be zero in a totally unbiased system. We observed it to be very small, but statistically significant (correlation = -0.12, p-val = 0.002) (Fig. S52).

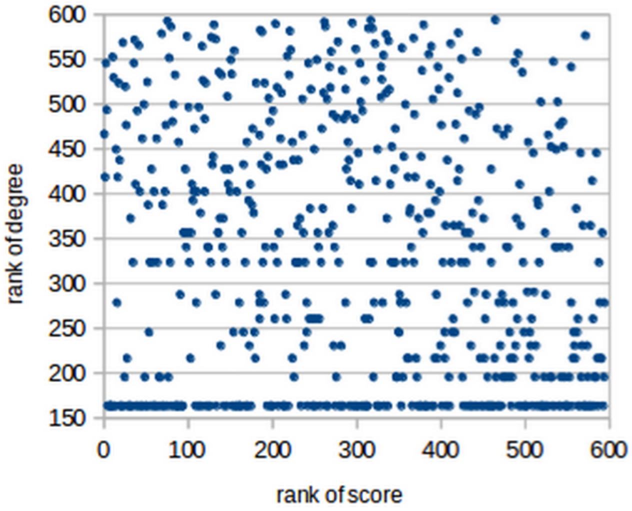

Figure S52: **The plot of the rank of the score of false genes versus the rank of their degree on the signaling network.** Low-rank of score means more significant and high-rank of degree means more connected.

Even though the bias is small, it needs to be explained. When we repeat the calculation for the results of the simulation in which no networks were used, the correlation becomes insignificant (correlation = -0.03, p-val = 0.50), so the degree bias is really related to the usage of networks.

After an extensive investigation, we noticed that this bias is not due to insufficient penalization of high-degree genes, but it is due to the discrete nature of p-values and the unequal distribution of statistical power in the system. For a gene, the number of all possible p-values is bound with the number of possible overlap patterns in alterations. For some genes, this number of observed distinct overlap patterns is very low during randomized runs. Consider the two example distributions of member p-values in Figure S53 (null distribution of p' values in Figure 2 of the main article, for two different genes). These are extreme cases of both ends. The left one is close to the expected uniform distribution. It is plotted with 10000 randomizations, and p' got 1212 distinct values. The right one is also plotted using 10000 randomizations, but p' got only 15 distinct values, and the p' value of the most significant score is 0.17. This second gene is not capable of generating noise in the high significance region. The first gene has potential to be anywhere in the ranking of scores, but the second gene cannot be at the most significant regions of the ranking.

Figure S54 is a redraw of the plot in Figure S52, but this time the genes with low statistical power are shown with red. This plot shows that the density of the low-statistical-power genes is higher in the low-degree region, as there are 77 red dots below the horizontal 300 line, and 52 above it. This makes sense because low-degree genes are tested with less number of combinations of other genes, and this will result in less number of distinct p-values. If they also have a low alteration ratio, this would result in large gaps among possible p-values, and some empty region at the high significance part of the distribution.

To demonstrate that the cause of the bias is the unequal distribution of the statistical power, we re-calculated the correlation after removing the red genes from the analysis and re-ranking the blue genes. This made the correlation to reduce to -0.07 with p-value 0.13. If we further decrease the minimum possible p-value threshold from 0.05 to 0.01 (makes half of the genes red), the correlation becomes -0.04 with p-value 0.54.

So we conclude that our scoring metric is not biased toward high-degree genes, but there is a small bias in the distribution of statistical power against low degree genes in the system.

Similarly, our metric is not biased toward highly altered genes, but we are able to detect a very small but statistically significant bias in the results of the simulation on large dataset that do not use the signaling network. Again, we confirmed that this is due to the statistical power bias against less altered genes.

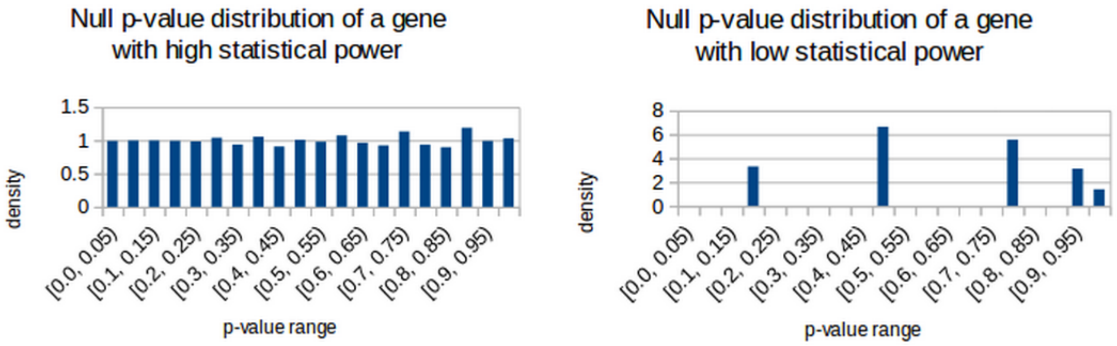

Figure S53: **Two example null distributions of member p-values for two different genes in simulations.** Left distribution belongs to a case where we have high statistical power, and the right distribution belongs to a case where we have low statistical power.

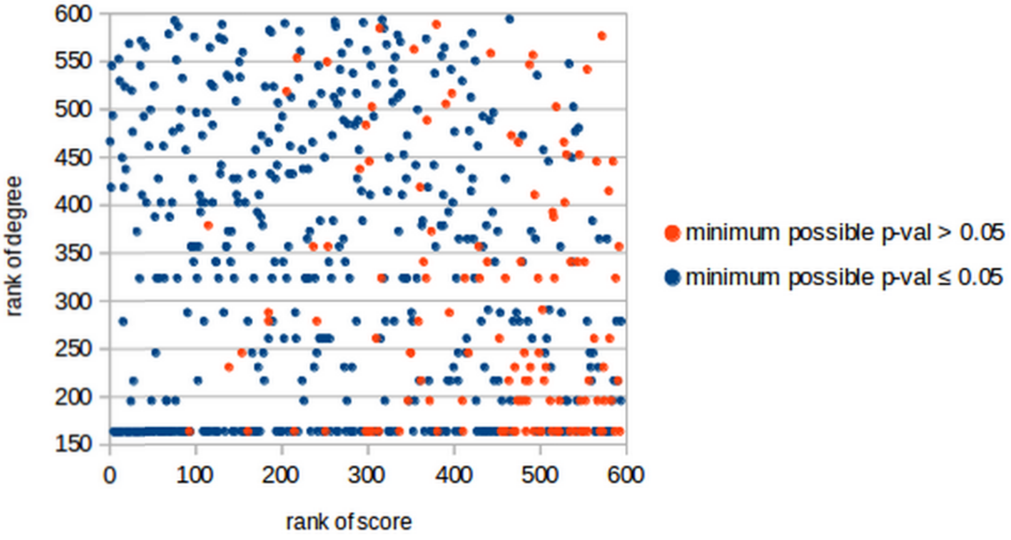

Figure S54: **Same plot as in Figure S52, but genes with low statistical power are marked.**

The frequent occurrence of *TP53* in our results is likely due to two reasons: its very high alteration frequency and biological centrality to multiple cancer processes. When a gene contains many alterations, as in the case of *TP53*, we have more statistical power and its mutex groups are more likely to be detected compared to the genes with few alterations. A mutex group with low coverage is likely to be not statistically significant for a low number of samples. This effect becomes negligible as the number of samples goes to infinity. In practice, however, we are operating with a very low number of samples – often a couple of hundred per cancer, compared to the complexity of statistical interactions we are trying to elucidate. This will change in the near feature as many cancer centers started to sequence patients on a routine basis. It will be extremely interesting to see how the landscape of results will change once we have tens of thousands of samples instead of hundreds.

Also biologically *TP53* is special, even among the other genes that are highly altered in cancer, such as *BRAF* or *KRAS*, in the sense that it is a central multiswitch controlling apoptosis, dna repair and cell cycle arrest. It controls the expression of a wide array of genes and downstream pathways. As a result, *TP53* status alone is considered as a subtype marker in many cancers. We believe that this cancer-specific centrality is the other primary reason for abundance of modules containing *TP53* in our results.

References

[1] Qiao, N., Huang, Y., Naveed, H., Green, C.D., Han, J.-D.J.: CoCiter: An efficient tool to infer gene function by assessing the significance of literature co-citation. PloS one **8**(9), 74074 (2013)

[2] Yeang, C.H., McCormick, F., Levine, A.: Combinatorial patterns of somatic gene mutations in cancer. The FASEB Journal **22**(8), 2605–2622 (2008)

[3] Vandin, F., Upfal, E., Raphael, B.J.: De novo discovery of mutated driver pathways in cancer. Genome Research **22**(2), 375–385 (2012)

[4] Zhao, J., Zhang, S., Wu, L.Y., Zhang, X.S.: Efficient methods for identifying mutated driver pathways in cancer. Bioinformatics (2012)

[5] Leiserson, M.D., Blokh, D., Sharan, R., Raphael, B.J.: Simultaneous identification of multiple driver pathways in cancer. PLoS computational biology **9**(5), 1003054 (2013)
